# A Liquid Ge(IV) Precursor for Low Temperature Plasma Enhanced Atomic Layer Deposition of Germanium Oxide Thin Films

**DOI:** 10.1002/smll.202511982

**Published:** 2026-02-19

**Authors:** Florian Preischel, Karl Rönnby, Martin Wilken, Jean‐Pierre Glauber, Samuel Froeschke, Detlef Rogalla, Thomas Gemming, Alexey A. Popov, Peter Dement, Michael Nolan, Anjana Devi

**Affiliations:** ^1^ Inorganic Materials Chemistry Ruhr University Bochum Bochum Germany; ^2^ Leibniz Institute for Solid State and Materials Research Dresden Germany; ^3^ Tyndall National Institute Lee Maltings University College Cork Cork Ireland; ^4^ RUBION Ruhr University Bochum Bochum Germany; ^5^ Fraunhofer Institute for Microelectronic Circuits and Systems (IMS) Duisburg Germany; ^6^ Chair of Materials Chemistry Dresden University of Technology Dresden Germany

**Keywords:** atomic layer deposition, density functional theory, main group elements, precursor design, thin films

## Abstract

Germanium oxide thin films are promising for advanced applications such as microelectronics, optoelectronics, high‐power electronics, optics, and biomedical uses. However, scalable and controlled low‐temperature synthesis of GeO_2_ thin films via atomic layer deposition (ALD) is limited by the small range of available Ge precursors. We introduce monomeric tetrakis‐3‐(*N*,*N*‐dimethylamino)propyl germanium(IV) [Ge(DMP)_4_] as a promising Ge precursor. It is non‐pyrophoric, thermally stable, and liquid, and can be obtained in high purity on a multigram scale through an industrially feasible synthesis. Using density functional theory (DFT) and mass spectrometry (MS), we rationalize the coordination environment and identify a feasible chemisorption pathway, indicating a high reactivity of the precursor. Subsequently, [Ge(DMP)_4_] was employed in low‐temperature plasma‐enhanced ALD (PEALD) over a wide temperature range from 40°C to 240°C, yielding smooth, uniform germanium oxide films. Rapid and homogeneous nucleation leads to dense films with sub‐nanometer thickness. By adjusting the deposition temperature and plasma duration, the film composition could be readily tuned from GeO_2_ to sub‐stoichiometric GeO_x_. These findings establish [Ge(DMP)_4_] as an effective, scalable precursor for low‐temperature ALD of GeO_2_, emphasizing the critical role of precursor chemistry in ALD process development.

## Graphical abstract

Utilizing the 3‐(*N*,*N*‐dimethylamino)propyl (DMP) ligand, the new liquid Ge(IV) precursor [Ge(DMP)_4_] has been developed, which possesses adequate volatility, reactivity, and exceptional thermal stability. This precursor is capable of low‐temperature processing of GeO_2_ and GeO_x_ with tunable film composition and sub‐nm thickness control in a range from 40°C to 240°C via plasma‐enhanced atomic layer deposition (PEALD).

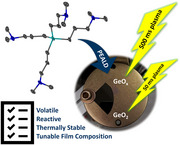

## 1 Introduction

Germanium dioxide (GeO_2_, also known as germania) thin films are highly valued in advanced electronics and memory devices [1]. The high permittivity of GeO_2_ (*k* ∼6) [2] along with its ultrawide bandgap (UWBG) ranging from 4.3 to 6.0 eV [3, 4, 5], and its compatibility with both Si and Ge substrates, makes it an important material for next‐generation complementary metal‐oxide semiconductor (CMOS) technologies. In particular, it holds potential as a UWBG semiconductor for high‐frequency and high‐power electronics due to its high carrier mobility [1, 6, 7]. Additional applications include anti‐reflective coating (ARC) in solar cells [8], functional layers in gas sensors [9, 10], and emerging applications in the biomedical sector, where GeO_2_ is explored for biocompatible antibacterial materials [11, 12] and bioactive glasses as bone implants [13, 14]. Because of the thermal sensitivity of medical substrates, these applications depend on low‐temperature processing.

Notably, Ge offers higher carrier mobilities than Si and is thus considered a potential successor for semiconducting devices. However, the practical implementation of Ge is hampered by challenges in forming a robust passivation layer. Controlled deposition of GeO_2_ as a complementary dielectric layer with a defined interface could significantly advance Ge‐based devices [2, 6, 15, 16, 17]. Beyond stoichiometric GeO_2_, oxygen‐deficient oxides (i.e., GeO and GeO_x_ with x < 2) exhibit distinct optical and electronic properties, further expanding the material's versatility [18, 19, 20, 21, 22, 23]. Oxygen‐deficient GeO_x_ serves as an active layer in resistive‐switching memory [24, 25, 26], dark‐current suppressant in Ge photodiodes [27], and stabilization layer for high‐capacity anodes in lithium‐ion batteries [28, 29, 30]. Amorphous GeO_x_ has also been investigated as a high‐capacity anode in sodium‐ion batteries [31, 32]. In addition, its optical constants (n, k) vary systematically with the degree of oxygen vacancies [33]. Accordingly, synthesis routes that deliver GeO_2_ and GeO_x_ with tunable stoichiometry provide a direct way to modify refractive index, switching behavior, and electronic characteristics, thereby enabling precise tuning of device properties.

Beyond applications that utilize the functional properties of GeO_2_, its structural characteristics open another interesting field of research. Recently, interest in 2D germanium monochalcogenides for optoelectronics has grown, with GeS, GeSe, and GeTe having been synthesized by atomic layer deposition (ALD) [34]. Furthermore, the exploration of 2D bilayer GeO_2_ could yield a new material for gas‐separation membranes. While such a bilayer structure has been experimentally demonstrated and analyzed for the analogous SiO_2_ [35, 36, 37, 38], it has so far been investigated mostly theoretically for GeO_2_ [39, 40, 41].

Only recently have a few studies described its successful preparation [42, 43]. Like bilayer silica, bilayer germania features molecular‐sized pores as an inherent structural element but with a different structural arrangement and pore size distribution [43, 44]. In the case of silica, the scalable fabrication of a freestanding membrane from a SiO_2_ bilayer grown by plasma‐enhanced ALD (PEALD) was previously demonstrated. The inherent pores enabled selective gas separation [45, 46, 47], eliminating the need for artificial perforation or modification, as required for membranes based on, e.g., graphene [48, 49]. Realizing an analogous freestanding GeO_2_ bilayer structure could enable the fabrication of a new membrane material with tailored gas selectivity. However, the stability of germania and the related bilayer structure has been reported to be problematic [39, 40, 44], making the fabrication of a stable freestanding bilayer a challenge. To enable these applications of germania and related materials, a scalable bottom‐up fabrication method is necessary. ALD is particularly well‐suited for this purpose, enabling self‐limiting deposition of high‐quality thin films with precise control over composition and thickness. Due to its surface‐bound growth mode, ALD further enables conformal deposition over nanostructures and complex device architectures [50, 51, 52]. PEALD typically allows deposition at lower temperatures, thereby broadening the process window to temperature‐sensitive applications such as flexible electronics [53, 54].

However, the development of ALD processes strongly depends on the availability of suitable precursors that combine high thermal stability with adequate volatility and reactivity towards the desired substrate and co‐reactant. Ideally, an ALD precursor should be liquid at room temperature to facilitate handling and maintain a constant evaporation rate [55, 56]. Due to the challenges of meeting these requirements, the development of GeO_2_ ALD processes has so far been limited to only a few precursors. An overview of published precursors and their process details is given in Table 1. These include the homoleptic [Ge(NMe_2_)_4_] [57, 58] as well as heteroleptic variants where two of the monodentate amide ligands are substituted by one variation of an ethylenediamine ligand in [Ge(NMe_2_)_2_(NH*^i^* Pr(CH_2_)_2_NH*^i^* Pr)] and [Ge(NMe_2_)_2_(NH*^t^* Bu(CH_2_)_2_NH*^t^* Bu)] [18]. Additionally, the *n*‐butoxide‐based precursor [Ge(O*^n^* Bu)_4_] has been used by Yoon et al. [57], and another precursor features the chelating 1,2‐bis[(2,6‐diisopropylphenyl)imino] acenaphthene (dpp‐BIAN) ligand in [Ge(dpp‐BIAN)] [4]. All these processes require the strongly oxidizing ozone (O_3_) as a co‐reactant and generally high temperatures, which can cause damage to sensitive substrates. This can adversely affect applications that rely on a well‐defined interface between the substrate and the deposited material, as well as the need for precise stoichiometric control. Only recently have advances been made with alternative co‐reactants. For instance, [Ge(thd)Cl] (thd = 2,2,6,6‐tetramethyl‐3,5‐heptanedione) has been used in combination with H_2_O_2_ by Choi et al. [59], whereas Hultqvist et al. [60], as well as Shin et al. [61] employed [Ge(NMe_2_)_4_] respectively with H_2_O and an O_2_ plasma as the co‐reactant. Although [Ge(O*^n^* Et)_4_] has been used with H_2_O, that study focused on the electrical properties of the deposited material, and details on the ALD process characteristics were not reported [62].

**TABLE 1:** Overview of Ge precursors and process parameters for the deposition of GeO_2_ by ALD.

Precursor	Co‐reactant	T_dep_ (°C)	GPC (Å)	Refs.
[Ge(dpp‐BIAN)]	O_3_	185–225	0.50	[4]
[Ge(NMe_2_)_4_]	O_3_	75–230	0.45	[57]
	O_3_	150–300	0.53	[58]
	H_2_O	120	0.30	[60]
	O_2_ plasma	70–250	0.55–0.75	[61]
[Ge(NMe_2_)_2_(*N*,*N`*‐(*^i^* Pr)_2_‐en]	O_3_	200–320	0.40	[18]
[Ge(NMe_2_)_2_(*N*,*N`*‐(*^t^* Bu)_2_‐en]	O_3_	200–320	0.31	[18]
[Ge(O*^n^* Bu)_4_]	O_3_	180–330	0.30	[57]
[Ge(thd)Cl]	H_2_O_2_	150–400	0.27	[59]
**[Ge(DMP)_4_]**	O_2_ plasma	40–240	0.10–0.55	This work

The hitherto reported precursors still require elevated deposition temperatures to deposit GeO_2_ thin films, except for [Ge(NMe_2_)_4_]. However, the lowest temperature achieved with this precursor is still 70°C [61], while it decomposes at elevated temperatures [57], indicating limited thermal stability. To address this issue and develop a new Ge precursor that is both processable at low temperatures and thermally stable, we utilized the 3‐(*N*,*N*‐dimethylamino)propyl (DMP) ligand, which has been proven to be a powerful tool toward precursors that enable low‐temperature (PE)ALD [63, 64, 65, 66].

Herein, we report the successful synthesis and detailed characterization of a new Ge precursor, tetrakis(3‐(*N*,*N*‐dimethylamino)propyl) germanium [Ge(DMP)_4_], and its effective use in low‐temperature PEALD of GeO_x_ thin films, employing O_2_ plasma as the co‐reactant. It is well known that plasma‐based processes offer an alternative to ozone, enabling low processing temperatures and greater flexibility in tuning film properties via plasma parameters (specifically, plasma power and exposure time) [53, 67, 68].

A systematic investigation was conducted to examine the influence of plasma parameters on the stoichiometry and composition of the films deposited. First principles density functional theory (DFT) calculations were performed to gain further insight into the structure and deposition chemistry of this new precursor.

## 2 Results and Discussion

### 2.1 Synthesis and Characterization of [Ge(DMP)_4_]

**SCHEME 1:**
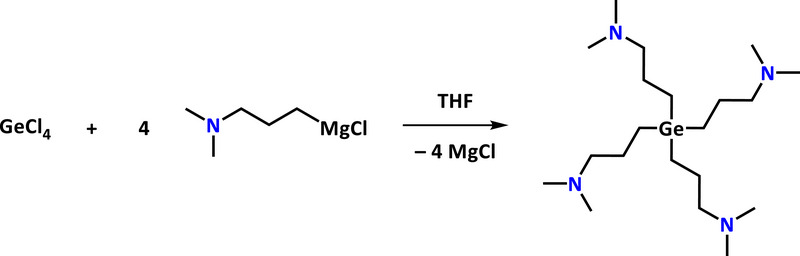
Synthesis of [Ge(DMP)_4_] via a salt metathesis reaction.

Germanium alkyl complexes are readily prepared via Grignard reactions [69, 70], and we adopted this straightforward approach to synthesize [Ge(DMP)_4_] in multigram quantities. For this, we used the freshly prepared Grignard reagent [(DMP)MgCl], synthesized according to literature‐reported procedures [66], and employed it in a salt metathesis reaction with GeCl_4_, as shown in Scheme 1. Following the same method, Zickgraf and co‐workers previously could only obtain the hydrochloride salt of [Ge(DMP)_4_] [71]. In our case, small but distinct changes in the workup led to the formation of the desired [Ge(DMP)_4_] as confirmed by analysis of the compound. The target compound is a colorless, clear liquid and was isolated by distillation at 100°C, yielding 16.2 g (62 %) of high quality and purity. [Ge(DMP)_4_] remains a liquid even at temperatures well below room temperature, which is highly beneficial for its application as a precursor in chemical gas phase deposition methods and suggests reduced intermolecular interactions between the individual molecules.

**FIGURE 1:**
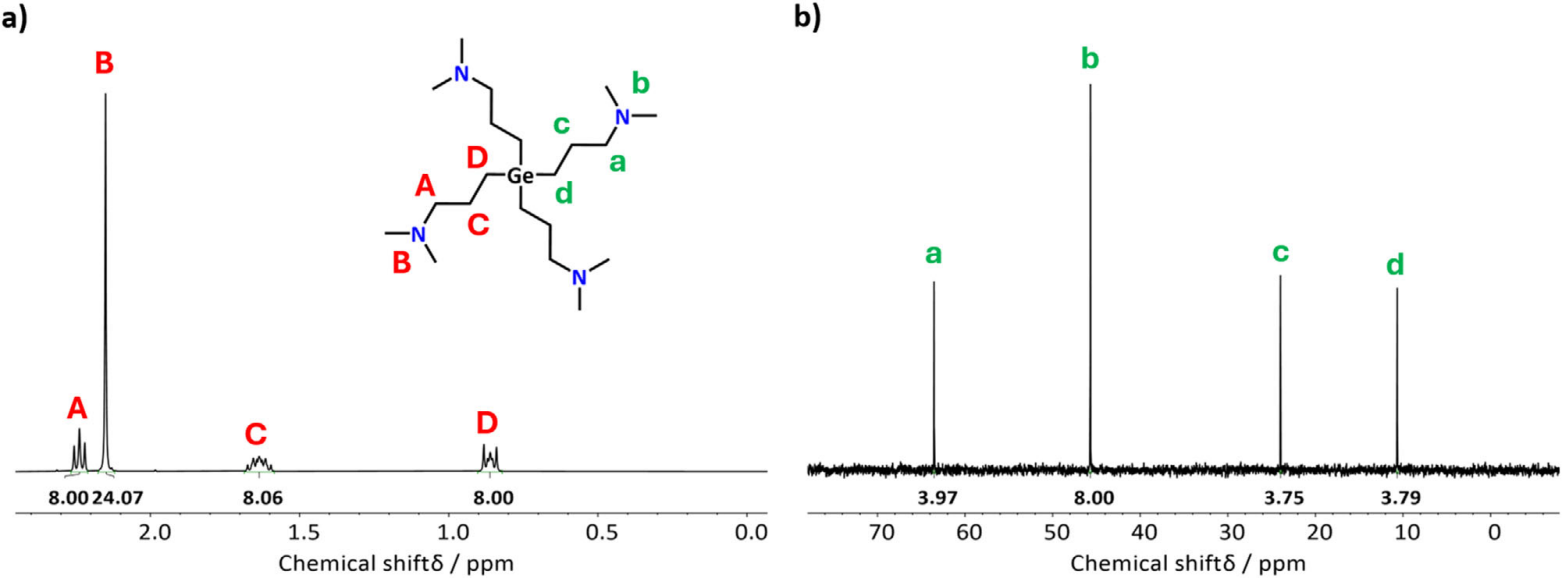
(a) ^1^H and (b) ^13^C NMR spectra of [Ge(DMP)_4_] with peak assignments.

The spectroscopic purity of the obtained compound was confirmed by ^1^H and ^13^C NMR spectroscopy, which showed the expected signals. This is shown in Figure 1 with an assignment of the detected signals to the respective atoms in [Ge(DMP)_4_]. A more detailed description of the NMR spectra is provided in the Supporting Information, alongside 2D NMR spectra (Figures S2 and S3), which further confirmed the proposed assignments. Interestingly, the ^1^H NMR shows an unusual splitting of the protons from the two CH_2_ groups adjacent to the metal center (a magnified view of these regions is shown in Figure S1), which cannot be solely explained by *J_HH_* coupling.

A similar splitting was previously observed for [Sn(DMP)_4_] [64], indicating that the Ge and Sn compounds possess comparable atomic structures. Since a non‐coordinating bonding motif of the DMP ligand to the Sn center was proposed, resulting in only Sn─C bonds being present, it is likely that analogously, [Ge(DMP)_4_] features only Ge─C bonds, as well. To further verify this assumption, complementary analyses were conducted as discussed below. Nonetheless, the absence of any impurity signals in the NMR spectra, alongside well‐matching elemental analysis (EA) values for C, H, N, and Ge (presented in Table S1), proves the successful synthesis of the target compound [Ge(DMP)_4_] with exceptional spectroscopic purity.

To assess the behavior of [Ge(DMP)_4_] when exposed to air, the pure compound was placed in open air and monitored over an extended period. As captured by a series of images (Figure S4), there was no visible change to the naked eye, and notably, no pyrophoric reaction occurred. This highlights that [Ge(DMP)_4_ is non‐pyrophoric and can be handled safely, which is crucial for scaling up toward industrial applications.

**FIGURE 2:**
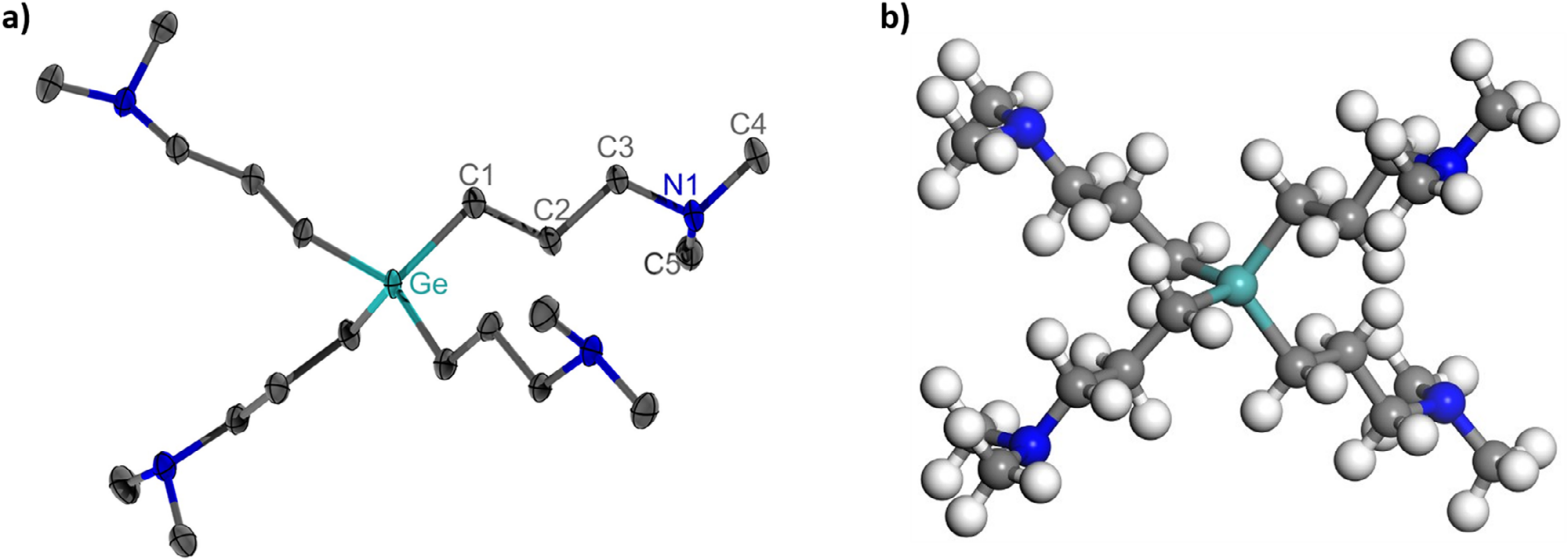
(a) SC‐XRD and (b) DFT structures of [Ge(DMP)_4_]. In the SC‐XRD structure, atoms are shown as ellipsoids with 50% probability, and hydrogen atoms are omitted for clarity. Color scheme: turquoise indicates Ge, grey C, blue N, and white H.

For direct structure determination, single‐crystal X‐ray diffraction (SC‐XRD) is essential. However, [Ge(DMP)_4_], being a liquid with a melting point of approximately −50°C (as observed during crystallization experiments), made it difficult to obtain suitable crystals, requiring storage of the compound in dry ice. After several attempts, we succeeded by using specific crystallization conditions described in the Experimental Section. Once successfully crystallized, SC‐XRD revealed a monomeric structure of [Ge(DMP)_4_] in the solid state with a monoclinic crystal system, as shown in Figure 2a. Notably, this differs from previously obtained structures of precursors such as [Al(N*^i^* Pr_2_)_2_(DMP)] [63, 72], [Zn(DMP)_2_] [65, 73] or [Mg(DMP)_2_] [66] where the DMP ligand forms a five‐membered ring through coordination of the amine group to the metal center. Instead, with four ligands surrounding the metal, each DMP ligand is forced to bind only to the Ge center via its terminal carbon atom, similar to the structure proposed for [Sn(DMP)_4_] [64]. This further highlight the structural relationship between the Ge and Sn compounds, as was inferred from their similar ^1^H NMR spectra. Conversely, this implies that [Sn(DMP)_4_] likely adopts a structure very similar to that of [Ge(DMP)_4_], where four DMP ligands coordinate to the metal center, resulting in a saturated electronic configuration that prevents the formation of dative bonds and the characteristic chelating bonding motif. Additionally, the steric hindrance from multiple DMP ligands appears to effectively suppress intermolecular interactions, thereby promoting the formation of monomeric complexes.

Consequently, the Ge center in [Ge(DMP)_4_] is coordinated by four atoms, forming a saturated eight‐electron complex with a highly symmetric structure (Figure 2a) where the bond angles vary only between 107.48 and 110.66 , closely matching the ideal tetrahedral angle of 109.5 . This is indicated by τ_4_ [74] as well as τ_4_’ [75] values of 0.98, representing an almost ideal tetrahedral coordination geometry around the Ge. Such high symmetry is beneficial in reducing intermolecular interactions, explaining the low melting point of the Ge compound. The structure is further visualized in Figure S6, which presents an alternative perspective, showing the coordination tetrahedron around the Ge center. Similarly, the four Ge─C bonds are almost identical with bond lengths of 1.966 and 1.970 Å. An overview of the bond lengths and angles around the Ge center is provided in Table S2, and crystallographic details are summarized in Table S3. Interestingly, the bond lengths and angles are comparable to homoleptic Ge aryl complexes such as [Ge(Ph)_4_] or [Ge(*o*‐tolyl)_4_] [76], despite the different inductive effect of the aryl versus alkyl ligands on the Ge─C bond. Similarly, average Ge─C bond lengths of 1.995 and 2.011 Å were recently reported for Ge(CF_3_)_4_ and Ge(C_2_F_5_)_4_, respectively, using in situ crystallization [77]. Yet, these fluorinated complexes adopt a more distorted tetrahedral arrangement, which may be explained by electrostatic repulsion between the fluorine atoms and the altered polarization of the fluorinated alkyl chains.

To the best of our knowledge, no crystal structures of homoleptic Ge(IV) complexes with non‐fluorinated alkyl ligands have been reported in the literature so far. This can likely be attributed to the low melting points around −90°C of Ge(IV) alkyls such as GeMe_4_ [70, 78, 79, 80]. The structure of [Ge(DMP)_4_]–which could be studied here using advanced crystallization techniques and careful handling at low temperatures–thus represents the first reported SC‐XRD structure of a non‐fluorinated homoleptic Ge(IV) alkyl species. Similarly, the SC‐XRD structure of the liquid [Sn(DMP)_4_] has not yet been determined, so it is not possible to compare the crystal structure of [Ge(DMP)_4_] with the directly related alkyl complexes. The molecular structure of [Ge(DMP)_4_] was therefore investigated using DFT (Figure 2b). The Ge center is in an almost perfect tetrahedral arrangement, with bond angles of 109.47 ° and equal Ge─C bonds with a length of 1.971 Å, which agrees very well with the SC‐XRD structure.

To evaluate whether a heteroleptic Ge complex, where the DMP ligands are partially replaced with less sterically demanding ligands, can facilitate the formation of the chelating bonding motif of DMP, DFT calculations of [Ge(DMP)_2_(NMe_2_)_2_] and [Ge(DMP)_2_Cl_2_] were performed. The heteroleptic complexes exhibit two possible bonding modes: monodentate binding of DMP to Ge through the carbon atom or chelation via an additional dative bond from the nitrogen. The geometry relaxations indicate that both forms are stable (Figure S7). In the chelated structure, the Ge atom adopts an octagonal geometry with four covalent bonds to the carbon of DMP and to Cl or the amide group, and two coordinating bonds to the nitrogen of DMP. The covalent Ge─C bonds are nearly the same length in both complexes, 2.011 Å for [Ge(DMP)_2_(NMe_2_)_2_] and 1.988 Å for [Ge(DMP)_2_Cl_2_], while the dative Ge─N bonds are longer for [Ge(DMP)_2_(NMe_2_)_2_] (2.42 Å) compared to [Ge(DMP)_2_Cl_2_] (2.31 Å). In the non‐chelated form, the Ge centers adopt slightly distorted tetrahedral geometries.

The non‐chelated Ge─C distances are consistent for both complexes, measuring 1.964 Å for [Ge(DMP)_2_(NMe_2_)_2_] and 1.955 Å for [Ge(DMP)_2_Cl_2_]. The non‐chelated form is more energetically favorable for the bulkier [Ge(DMP)_2_(NMe_2_)_2_] complex, with a stabilization of −183 kJ mol^−1^ compared to the chelated form. For [Ge(DMP)_2_Cl_2_], the chelated form is slightly more stable by −14 kJ mol^−1^. These findings support the idea that steric hindrance plays a significant role in why [Ge(DMP)_4_], with four bulky ligands, does not chelate, unlike other metal DMP complexes such as Mg(DMP)_2_, which has only two ligands and a coordination environment that allows chelation [66].

**FIGURE 3:**
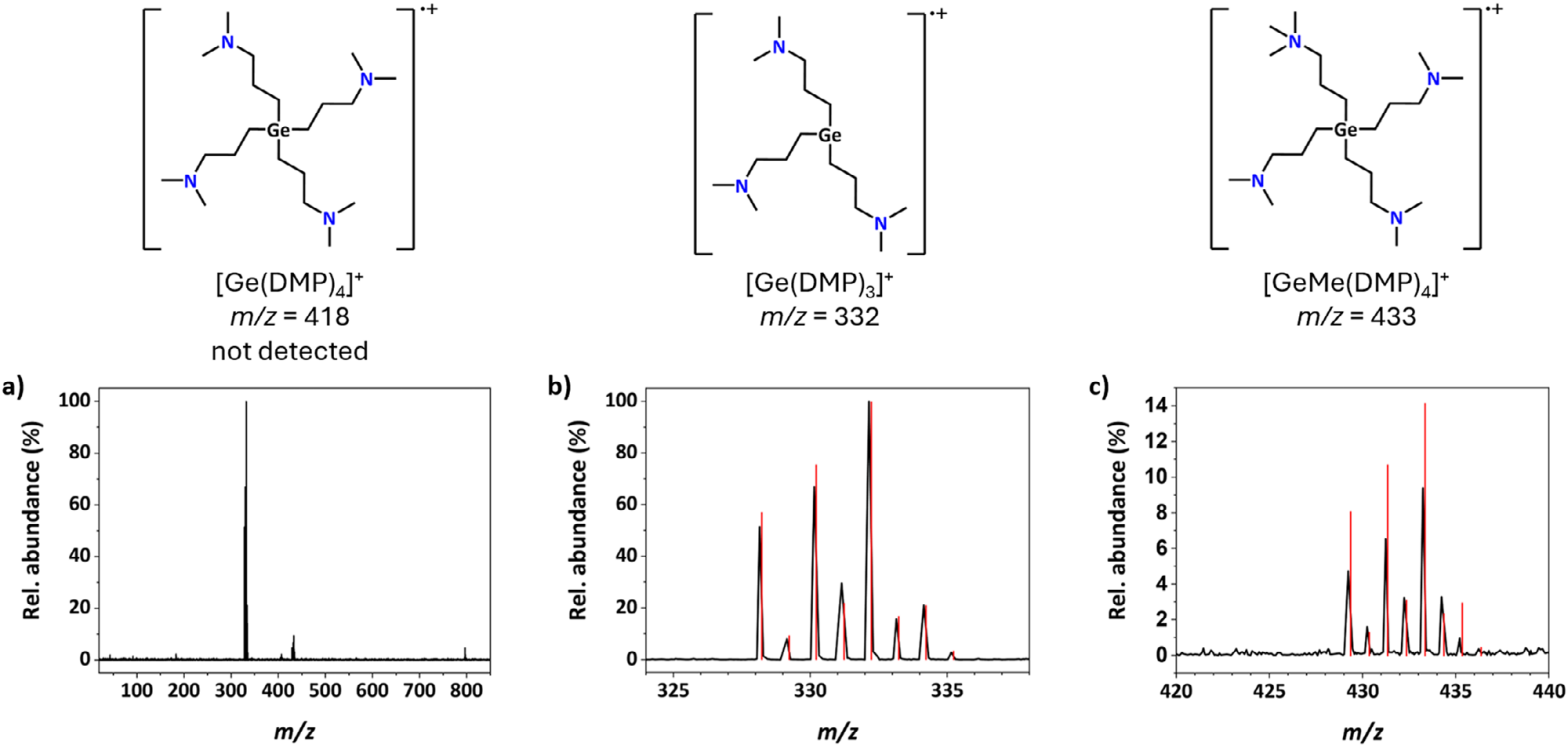
LIFDI MS spectrum of [Ge(DMP)_4_] showing (a) the whole spectrum, and in (b) and (c) a zoomed‐in view of relevant regions with an overlay of the expected isotope pattern in red and a structure of the corresponding Ge species on top.

For further characterization and structural clarification of [Ge(DMP)_4_], liquid injection field desorption ionization (LIFDI) MS was conducted. While the [M]^+^ peak was not detected, a [Ge(DMP)_3_]^+^ fragment (*m/z* = 332, 100 %) and a smaller signal that could be assigned to [GeMe(DMP)_4_]^+^ (*m/z* = 433, 9.4 %) were seen with matching isotope patterns, as can be seen in Figure 3. Because the Ge center in [Ge(DMP)_4_] is saturated with symmetric, tetrahedral coordination, the extra [CH_3_]^+^ group in [GeMe(DMP)_4_]^+^ is naturally attributed to one of the amine groups. However, it is not possible to determine the structure of this fragment with certainty based on the available data. Nevertheless, the observed signals suggest that one DMP ligand is easily cleaved, partially forming a new species even under the relatively mild LIFDI ionization conditions. The loss of one DMP ligand aligns with DFT calculations of the [Ge(DMP)_3_] radical. This could be promising for ALD experiments, as the open coordination site may enhance the precursor's probability of chemisorption on the surface. To assess this ligand elimination in the gas phase, we followed our previous approach to compute ligand‐elimination energies and structures [81, 82]. The calculated energy cost to eliminate the first DMP ligand as a radical in the gas phase is 313 kJ mol^−1^. Although this gas‐phase ligand‐elimination energy is moderately high, ligand loss can be readily achieved under ALD conditions, thereby enabling chemisorption on a substrate. Further, the structure of the [Ge(DMP)_3_] radical after loss of one ligand was considered using DFT. Notably, chelation of the three remaining ligands to the Ge center is not observed. The Ge─C bonds slightly elongate to 1.981 Å, while the C─Ge─C bond angles stay nearly the same at 109.67 . This confirms the proposed structure of the detected LIFDI‐MS fragment and its ability to enhance chemisorption due to the open coordination site at the Ge center. At the same time, LIFDI‐MS showed no relevant signals that could suggest at the presence of dimeric or oligomeric species at higher *m/z* ratios, indicating a monomeric nature of [Ge(DMP)_4_] in the gas phase.

In summary, spectroscopically pure [Ge(DMP)_4_] was synthesized via a scalable method using a Grignard reagent. SC‐XRD and DFT studies indicate a monomeric structure that supports high volatility [83]. In its preferred liquid state, the compound shows promising features for ALD processing.

### 2.2 Thermal Evaluation of [Ge(DMP)_4_]

**FIGURE 4:**
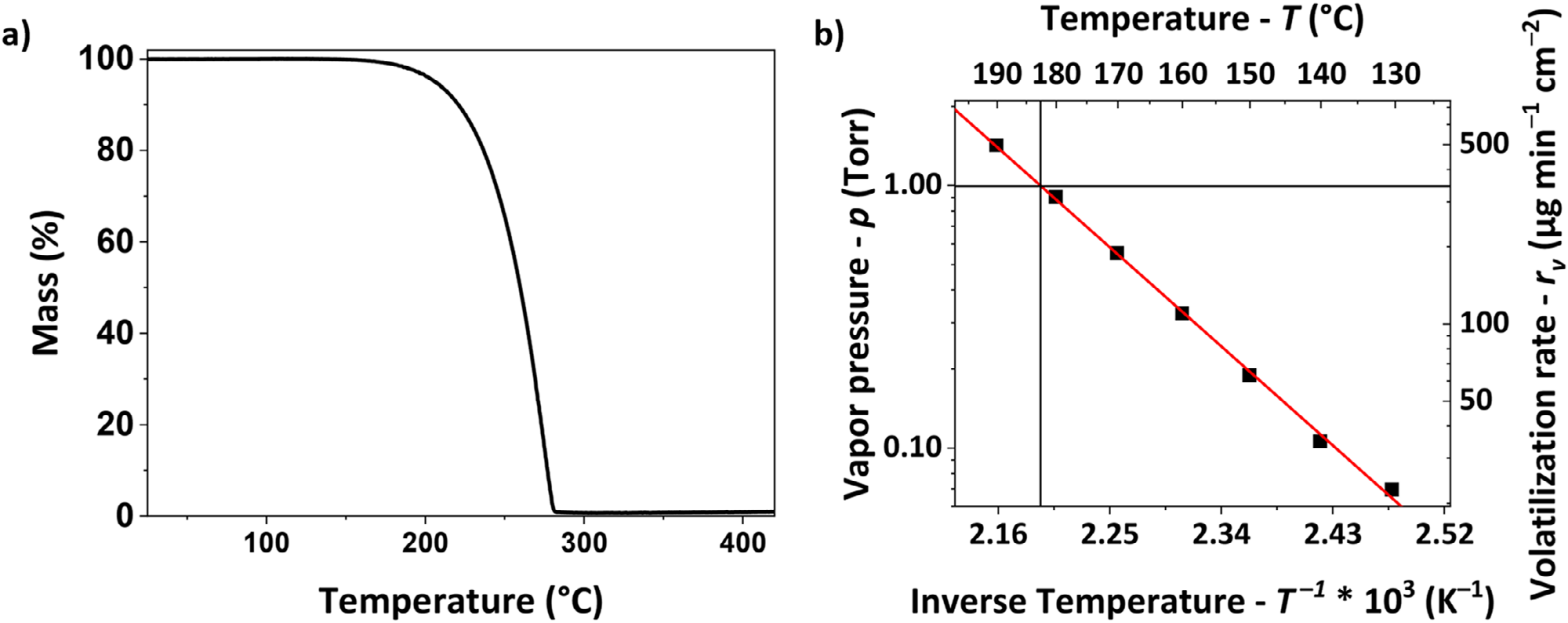
(a) TG curve of [Ge(DMP)_4_] and (b) Clausius–Clapeyron plot of [Ge(DMP)_4_] based on stepped iso‐TG data, where the intersection of the horizontal and vertical lines indicates *T_1Torr_*.

To further assess the suitability of [Ge(DMP)_4_] as a precursor, its thermal stability was studied using thermogravimetric analysis (TGA). The TG curve of [Ge(DMP)_4_], recorded at atmospheric pressure, showed an evaporation onset (1 % mass loss) at 178°C, followed by a single‐step weight loss with no residual mass (Figure 4a, data summarized in Table S4). These results suggest that [Ge(DMP)_4_] evaporates completely without decomposing under the specified measurement conditions. This evaporation behavior is similar to that of [Sn(DMP)_4_], which has a lower onset temperature of 158°C, despite its higher molecular weight [64]. However, the 1 % mass loss can be affected by the exact measurement conditions and, therefore, cannot be used as the sole parameter to assess precursor volatility. The step temperature (determined by the tangent method using the points of 1 % mass loss and the steepest mass loss) at 260°C further confirms the compound's excellent thermal stability, providing a broad temperature range suitable for evaporation.

Intrigued by this thermal stability, we examined the compound's thermal limits and performed an additional TG analysis at a higher mass loading and an increased heating rate of 20 K min^−1^ to expose the compound to higher temperatures before it fully evaporates [84]. The recorded curve (Figure S8) shows the same one‐step evaporation behavior as the original but, as expected, is shifted to higher temperatures. Despite exposure to these elevated temperatures, a residual mass of 0% is reached, and the step temperature of 304°C clearly demonstrates the exceptional thermal stability of [Ge(DMP)_4_] on the timescale of the TG measurement.

To further evaluate the volatilization behavior of [Ge(DMP)_4_], a stepped *iso*thermal TG was performed between 130°C and 190°C. Using the Langmuir equation, the vapor pressure of the analyzed precursor can be estimated for each *iso*thermal setpoint [85, 86]. With the resulting data points, the temperature at which a vapor pressure of one Torr (*T_1Torr_*) is achieved can be determined from a Clausius–Clapeyron diagram (Figure 4b) [86, 87]. For [Ge(DMP)_4_], *T_1Torr_* was determined to be around 183°C with a corresponding rate of volatilization (*r_v_*) of 329 µg min^−1^ cm^−2^. *T_1Torr_* is just slightly above the onset of evaporation, meaning that once a temperature sufficient for volatilization is reached, the compound already has a significant vapor pressure and evaporates readily at elevated temperatures. This is evidenced by the relatively narrow evaporation window and the steep decline observed in the TG curve.

Notably, the evaporation rates determined for [Ge(DMP)_4_] are higher than those measured for [Sn(DMP)_4_], which were obtained through separate static *iso*thermal measurements, e.g., 65 µg min^−1^ cm^−2^ versus 52 µg min^−1^ cm^−2^ at 150°C (Table S5). These results indicate that, despite the earlier onset of the Sn complex, [Ge(DMP)_4_] evaporates more readily at a given temperature, which correlates with its lower molecular mass and demonstrates the limited relevance of the 1 % mass loss to judge the volatility of a potential precursor. Due to the lower molecular mass of [Ge(DMP)_4_] compared to [Sn(DMP)_4_], this effect is more pronounced when the *r_v_* is converted to the Molar volatilization rate (*r_vm_*) (Table S5), which describes the number of molecules that evaporate from a given surface area. Besides *T_1Torr_*, *r_vm_* is therefore an essential thermal property of an ALD precursor, because a high number of molecules transported to the substrate surface is necessary to allow efficient saturation in each ALD cycle. From the detailed thermal analysis, it can be concluded that [Ge(DMP)_4_] exhibits a one‐step evaporation behavior with excellent thermal stability and volatilization rates suitable for an ALD precursor.

### 2.3 ALD Process Development and Compositional Analysis

**FIGURE 5:**
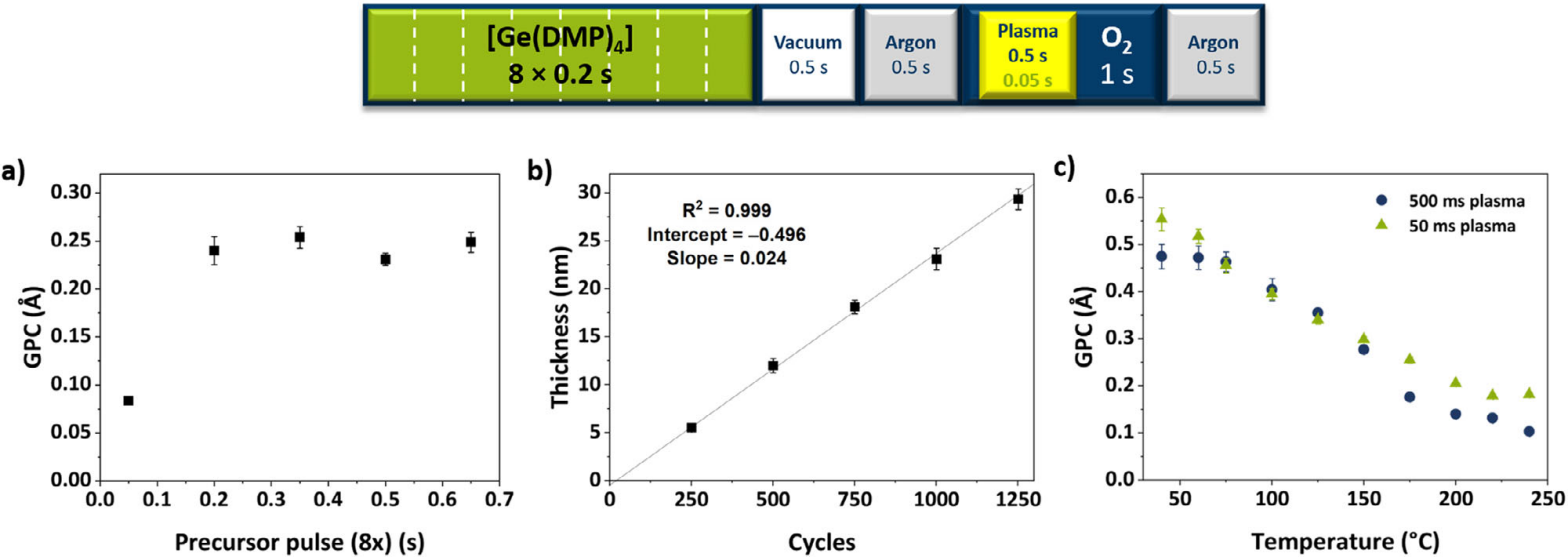
PEALD process development for GeO_x_ on Si substrates with a schematic representation of the optimized process at the top using [Ge(DMP)_4_] with an O_2_ plasma in terms of (a) precursor saturation, (b) linearity of the cycle number vs the thickness (both at 150°C) and (c) temperature dependency of the GPC at plasma times of 500 ms (blue circles) and 50 ms (green triangles).

Based on the promising physico‐chemical properties, PEALD of GeO_2_ was performed using [Ge(DMP)_4_] and O_2_ plasma. To develop the PEALD process, [Ge(DMP)_4_] was heated to 120°C with a substrate temperature of 150°C and an initial plasma‐pulse duration of 500 ms. Saturation was reached with a total precursor pulse duration of 1.6 s (eight consecutive pulses of 0.2 s, separated by 50 ms of vacuum) with a growth per cycle (GPC) of 0.24 Å (Figure 5a). The GPC remained constant with longer pulses, showing the self‐limiting nature of ALD growth. Compared with the PEALD process using [Sn(DMP)_4_], which was developed in the same reactor and under similar conditions but with a bubbler temperature of 140°C [64], [Ge(DMP)_4_] evaporates at a lower temperature, confirming its previously proposed higher volatility. Using the optimized pulse/purge sequence (depicted at the top of Figure 5) with cycle numbers between 250 and 1250, the thickness was found to increase linearly with the number of PEALD cycles, as expected for an ideal ALD process. From the slope of the linear fit (*R^2^* = 0.999), the GPC was determined to be 0.24 Å, consistent with the initial observation from the saturation study. The extrapolated linear fit also shows only a negligible offset, indicating that growth is neither hindered nor enhanced during the nucleation period. The determined GPC lies at the lower end of the range reported for ALD of GeO_2_ (Table 1). However, the short purge times required for the PEALD sequence enable comparably fast processing. For example, 500 cycles of the optimized process at 150°C (yielding a film thickness of 12 nm) are completed in approximately 34 min.

Reducing the plasma pulse duration stepwise from 750 to 50 ms increased the GPC from 0.23 to 0.30 Å (Figure S9a). It could be presumed that the elevated GPC results from incomplete combustion of the ligands when short plasma pulses are applied. This, however, is contradicted by consistently low impurity levels determined by Rutherford backscattering spectrometry in combination with nuclear reaction analysis (RBS/NRA), (Table S6). The composition, in terms of the O/Ge ratio, provides further insight and shows a correlation with the film density that is induced by the O_2_ plasma (Figure S9a). For plasma pulses between 750 and 250 ms, O/Ge ratios of 1.2 to 1.4 were determined, indicating a significant contribution of sub‐valent Ge^II^ or Ge^0^ species and coinciding with higher film densities up to 4.8 g cm^−3^–exceeding the typical 4.3 g cm^−3^ of hexagonal GeO_2_. In contrast, when the plasma pulse was shortened to 100 and 50 ms, the O/Ge ratio abruptly increased to 2.0, matching the stoichiometric formula of GeO_2_. This shows that even a 50 ms plasma pulse is sufficient to fully oxidize the chemisorbed precursor species to form stoichiometric GeO_2_. Under these conditions, the density settled at 3.9 g cm^−3^. As the areal density of Ge atoms detected by RBS mainly remained unchanged, variations in the O/Ge ratio primarily originate from changes in O incorporation as a direct consequence of the O_2_ plasma exposure duration. Thus, it can be concluded that the plasma dictates different growth modes–thereby influencing the GPC, oxidation state, and density of the deposited films.

To further investigate this phenomenon and examine the temperature dependence of the process, two temperature‐dependent studies were conducted with plasma pulse times of 500 and 50 ms, respectively (Figure 5c). Using 500 ms plasma pulses, variation of the substrate temperature revealed different growth regimes: After a constant initial regime from 40°C to 80°C, the GPC decreased continuously with increasing substrate temperature until the decline flattened above 200°C. On the other hand, the initial regime was missing when 50 ms plasma pulses were employed. Instead, the GPC decreased gradually from 40°C to 200°C and then remained nearly constant up to 240°C. Notably, even the films deposited at the lowest temperatures showed no discernible signs of condensation, despite *T_dep_* being well below the evaporation temperature of [Ge(DMP)_4_]. This was similarly observed in the SnO_2_ PEALD process with [Sn(DMP)_4_] [64], indicating the capability of both the DMP ligand and plasma‐based ALD processes for low‐temperature deposition.

**FIGURE 6:**
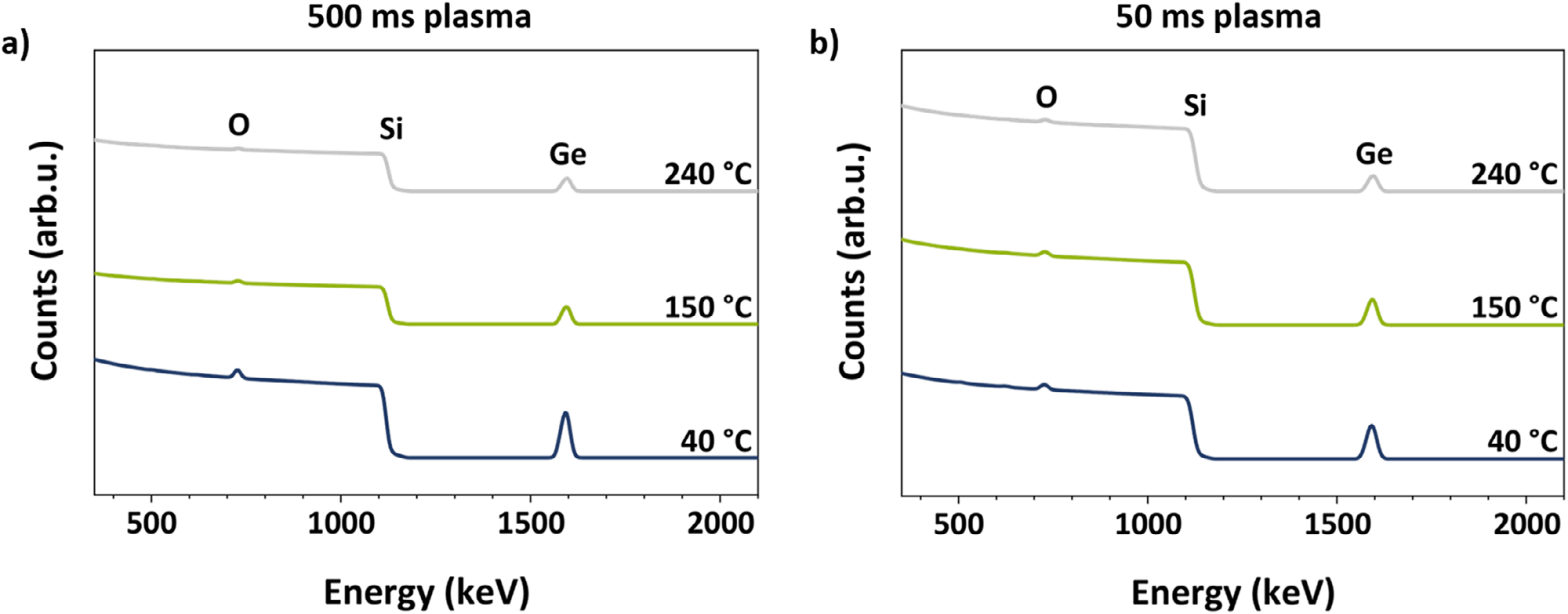
RBS plots of GeO_x_ thin films deposited on Si substrates at different temperatures with plasma pulse times of (a) 500 ms and (b) 50 ms.

As observed for different plasma pulse lengths, the density of the deposited layers followed a trend opposite to that of the GPC: it increased with temperature (Figure S9b,c), while maintaining similar steady‐state regimes. While increasing density was observed in both temperature studies, it was more pronounced and gradual in the study using 500 ms plasma pulses (3.9 g cm^−3^ at 40°C to 5.2 g cm^−3^ at 240°C). On the other hand, the density in the 50 ms plasma temperature study was more comparable to typical density values of other ALD deposited GeO_2_ films [61] and increased primarily in the temperature regime from 40°C (3.4 g cm^−3^) to 80°C (3.9 g cm^−3^) and then mainly remained constant. For both plasma pulse durations of 500 and 50 ms, the films deposited at 150°C were found to be amorphous as revealed by grazing incidence XRD (GI‐XRD) with no significant reflections in the 2ϴ range from 10° to 80° (Figure S10). Here again, the composition was examined with RBS/NRA, and representative RBS spectra are shown in Figure 6, clearly displaying the separate Ge peak as well as the O signal on top of the Si. The complete data is summarized in Tables S7 and S8. For thin films deposited with plasma pulses of 500 ms, the O/Ge ratio gradually changed from approximately 1.3 at 240°C to about 2.1 at 40°C, which is close to what is expected for GeO_2_. In contrast, an O/Ge ratio of around 2.0 was observed across the entire temperature range with 50 ms plasma, indicating the growth of GeO_2_.

Furthermore, the C and N impurities were consistently low at or below a few at.%, whereas only the deposition at 40°C with 50 ms plasma pulses showed significantly higher contamination levels (Tables S7 and S8). A similar trend of increased C and N with short plasma pulses at a low deposition temperature was observed for the [Sn(DMP)_4_] PEALD process. Likewise, this indicates that a minimum energy input from the plasma and deposition temperature must be reached to prevent condensation of precursor molecules and enable clean combustion of the ligands.

Altogether, plasma and deposition temperature collectively influence film growth: first, a high energy input from a combination of high temperature and long plasma pulses facilitates the formation of suboxide GeO_x_ species with decreased GPC but higher density. Secondly, a certain energy threshold needs to be reached by a suitable combination of temperature and plasma to fully enable clean combustion reactions. Therefore, the appropriate choice of deposition temperature and plasma pulse duration allows for the deposition of GeO_2_ or GeO_x_ with readily tunable composition.

The exact mechanism of Ge suboxide formation could not be experimentally determined within the scope of this study. However, it is well established that GeO_2_ on Ge(100) surfaces transforms to GeO at elevated temperatures, with GeO desorption occurring above 420°C [6, 88, 89]. Despite the different surface and that the investigated temperatures were higher than in our PEALD process, the additional energy input from the plasma might facilitate similar decomposition pathways. This is underlined by the thermal process reported by Choi et al., who employed [Ge(tmhd)Cl] with H_2_O_2_ as the co‐reactant [59]. The authors observed the opposite trend, with the GeO share decreasing from 11 % at 150°C to 5 % at 350°C. This indicates that, in our study, the formation of Ge^2+^ and Ge^0^ species at higher temperatures is driven by the plasma. This is consistent with the observation that O/Ge ratios significantly below 2 were observed only for longer plasma pulses, confirming that plasma is a crucial parameter that must be precisely controlled, with the added advantage of enabling tunable material properties. The influence of plasma is further validated by the findings by Shin et al. [61], who also reported a lower GPC paired with an increasing share of GeO at higher temperatures in a PEALD process using [Ge(NMe_2_]_4_] with O_2_ plasma and comparable pulse durations of 600 ms. Nevertheless, the dependence of the O/Ge ratio on temperature was less pronounced than in our process, suggesting that, in addition to the exact plasma‐source setup, precursor chemistry also plays a significant role in film formation.

Considering the effect of oxygen plasma on tuning the GeO_x_ composition, we investigated the formation of oxygen vacancies in GeO_2_ using DFT calculations. The structure of bulk and oxygen‐vacant GeO_2_ is shown in Figure S11. The formation energy was calculated using Equation (1), with a reference state in which a triplet oxygen radical (^3^O) interacts with GeO_2_ to release O_2_, resulting in GeO_x_.

(1) $$\begin{array}{c} \Delta E=\left(E_{Ge_{16}O_{31}}+E_{O_{2}}\right)\;-\left(E_{Ge_{16}O_{32}}+E_{O}\right) \end{array}$$

The oxygen vacancy formation energy is 0.80 eV relative to the ^3^O radical. Although the formation energy is endothermic, the energy cost is sufficiently low for vacancies to form during processing. If the plasma process supplies sufficient energy and oxygen radicals, this will generate oxygen vacancies and modulate the composition, consistent with the observation that the share of suboxide GeO_x_ species increases with temperature and plasma exposure.

During the PEALD process development, the long‐term thermal stability of [Ge(DMP)_4_] under operating conditions was investigated by analyzing the precursor after prolonged use at a bubbler temperature of 120°C. First, analysis by ^1^H NMR spectroscopy revealed no differences in peak positions, splittings, or integrals, and no peaks indicative of precursor decomposition were observed (Figure S5). In addition to the spectroscopic purity, the clean one‐step evaporation behavior of pristine [Ge(DMP)_4_] was also retained, as depicted in Figure S8. Overall, these results confirm the exceptional short‐ as well as long‐term thermal stability of the precursor.

### 2.4 X‐Ray Photoelectron Spectroscopy Analysis

**FIGURE 7:**
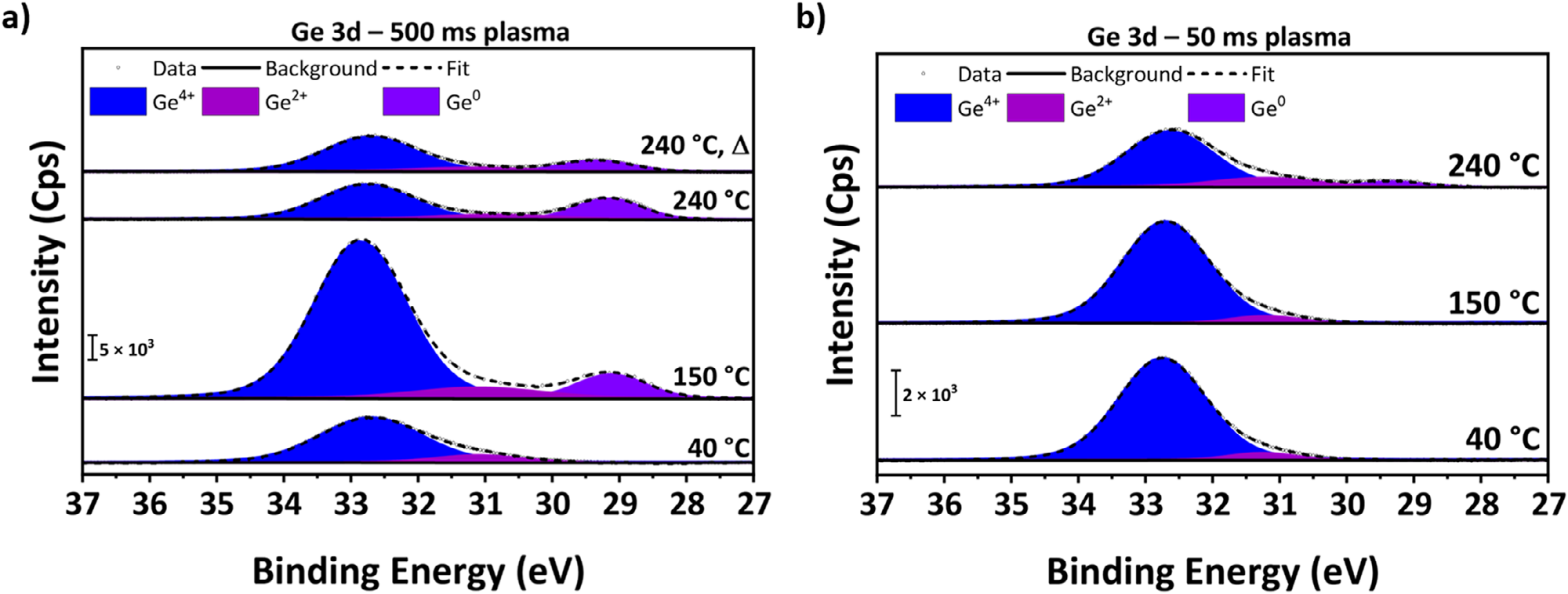
High‐resolution XPS spectra of the Ge 3d core level region of as‐deposited GeO_x_ thin films on Si at various temperatures. Samples in (a) were deposited with 500 ms plasma pulses, and the topmost sample was annealed after deposition for 20 min at 400°C under ambient conditions. Samples in (b) were deposited with 50 ms plasma pulses.

To further investigate the effects of plasma and temperature on composition and to obtain in‐depth insights into the Ge oxidation states in GeO_x_ thin films, X‐ray photoelectron spectroscopy (XPS) was performed on layers deposited at varying temperatures using 500 and 50 ms plasma pulses. As can be seen from a representative survey spectrum (Figure S12a), no signal was detected in the N 1s region, and the minor C 1s signal at 284.8 eV [90] is completely removed upon Ar^+^‐sputtering. Thus, the detected carbon can be attributed to adventitious carbon, and the films are impurity‐free. Most interesting is the Ge 3d core level region as it allows to distinguish between Ge^4+^, Ge^2+^, and Ge^0^ species. Notably, the sputter step significantly altered the film surface by completely reducing the Ge^4+^ content to Ge^2+^ and Ge^0^ (Figure S12b). Consequently, analysis of the films is restricted to the as‐introduced samples shown in Figure 7. The Ge^4+^ peak appears between 32.6 and 32.9 eV, the Ge^2+^ peak between 30.8 and 31.3 eV, and (where detected) the Ge^0^ signal is seen between 29.1 and 29.3 eV. These values are well in line with general literature and other ALD‐deposited GeO_x_ thin films [23, 57, 59, 91], just as for the O─Ge─O and O─Ge signals from the O 1s core level (Figure S13) [57, 59]. By comparing the deposition parameters, it is evident that the share of suboxides increases primarily with the plasma exposure and secondarily with the deposition temperature, as listed in Table S9. Here, it should be noted that the sample deposited at 150°C with 500 ms of plasma was measured using a different device, resulting in an overall increase in intensity in the diagram; however, the relative shares still follow the described trends.

For films deposited with 500 ms plasma, only the sample deposited at 40°C is completely free of Ge^0^, while with a reduced plasma exposure of 50 ms, a minor amount of Ge^0^ appears only at 240°C. From the shares of the different Ge species, the stoichiometry was calculated (Table S9), giving a range from GeO_1.95_ (40°C and 150°C with 50 ms plasma) to GeO_1.35_ (240°C with 500 ms plasma). The values determined by XPS follow the same trend as found by RBS/NRA. These findings confirm the prior assumption that excess plasma exposure is the main factor contributing to the formation of suboxides, highlighting the importance of process optimization in PEALD and providing a convenient means to tune the composition of the deposited material by combining plasma exposure and deposition temperature.

To further investigate the stability of the GeO_x_ film and the effect of temperature on the Ge oxidation states, the sample deposited at 240°C with 500 ms plasma was annealed at 400°C for 20 min under ambient conditions. The annealed sample was subjected to RBS/NRA (Table S7), revealing no change in the detected Ge areal density but a slight increase in the O areal density, resulting in a rise in the O/Ge ratio from 1.33 to 1.42. At the same time, XPS showed a significant reduction of Ge^0^, a slight decrease of Ge^2+^, and an increase in Ge^4+^ (Figure 7 and Table S9). Therefore, it can be concluded that Ge^0^ and Ge^2+^ react with O_2_ from the atmosphere to form Ge^4+^, while desorption of GeO does not occur. Otherwise, the areal density of Ge measured by RBS would be lower than that of the as‐deposited sample. This is consistent with prior findings that GeO desorption occurs above 420°C [6, 88, 89] and suggests that the annealing process proceeds analogously to the thermal oxidation of Ge. These initial findings are promising, as they demonstrate the stability of the thin film at elevated temperatures and suggest that the sub‐stoichiometric GeO_x_ films may be fully transformed to GeO_2_ by employing longer annealing times and/or a pure O_2_ atmosphere.

### 2.5 Optical Thin Film Characterization

To evaluate the effect of different GeO_x_ compositions on the optical properties of the thin films, samples deposited with 500 and 50 ms plasma pulses at different temperatures on Si substrates were investigated using spectroscopic ellipsometry and UV/Vis measurements. As summarized in Table S10, a clear correlation between the composition and the refractive index *n* is observed: films with O/Ge ratios of approximately 2 have a refractive index of 1.62–1.65 at a wavelength *λ* of 500 nm, as is expected for pure GeO_2_ [33]. On the other hand, *n* increases with the share of suboxide species in the films deposited with 50 ms plasma pulses at 240°C (O/Ge = 1.87, *n* = 1.82) and 500 ms plasma at 150°C (O/Ge = 1.42, *n* = 1.98), which is in line with the higher refractive indices of GeO and Ge [33]. Shin et al. recently reported a similar trend, where the refractive index increases with the GeO content in GeO_x_ thin films [61]. Notably, the sample deposited with 500 ms plasma pulses at 240°C has a refractive index of 1.66, despite its O/Ge ratio of 1.33. This discrepancy from the expected trend could not be explained within the scope of this preliminary optical characterization and requires verification in more detailed studies. Without this outlier, *n* follows a clear trend, demonstrating that the refractive index can be systematically tuned by adjusting deposition parameters, consistent with previous findings in the literature [33].

**FIGURE 8:**
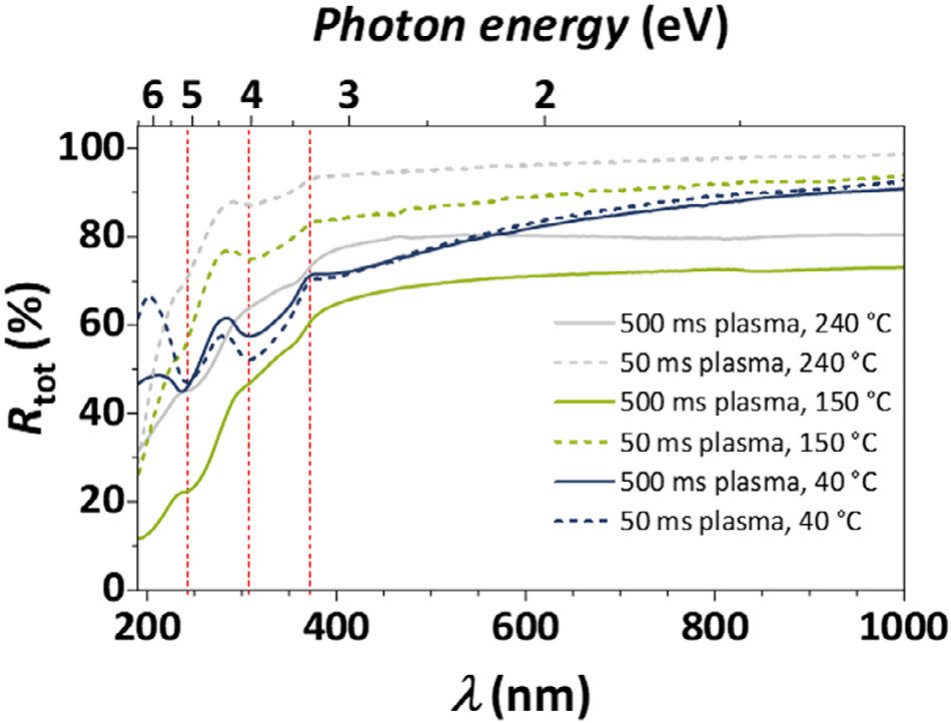
Total reflectance spectra of GeO_x_ thin films deposited on Si substrates at different temperatures with plasma pulse times of 500 ms (solid lines) and 50 ms (dashed lines). A bare Si substrate was used as a reference. Vertical, dashed red lines mark notable features.

Total reflectance UV/Vis measurements (Figure 8) show reflectance values of 90% or higher for samples with compositions close to GeO_2_ at longer wavelengths, consistent with the UWBG of GeO_2_ and thus its transparency to low‐energy light. The thin films deposited with 500 ms plasma pulses at 150°C and 240°C, however, exhibit lower reflectance values, which can be attributed to the increased proportion of suboxide species. At around 371 nm, the reflectance of all samples begins to decrease. Particularly, the samples deposited at 40°C show sub‐band features at 307 and 242 nm, which are significantly decreased in the samples deposited at 150°C and 240°C. These might be explained by defect states that occur in the GeO_x_ structure at lower deposition temperatures.

Overall, the initial optical characterization reveals promising ways to tune the refractive index and reflectance behavior of GeO_x_ thin films in the UV and visible ranges. Controlling the deposition temperature and plasma pulse duration thus allows for the growth of thin films with optical properties tailored to the intended application.

### 2.6 Morphological Thin Film Characterization and GeO_2_ Nucleation Studies

**FIGURE 9:**
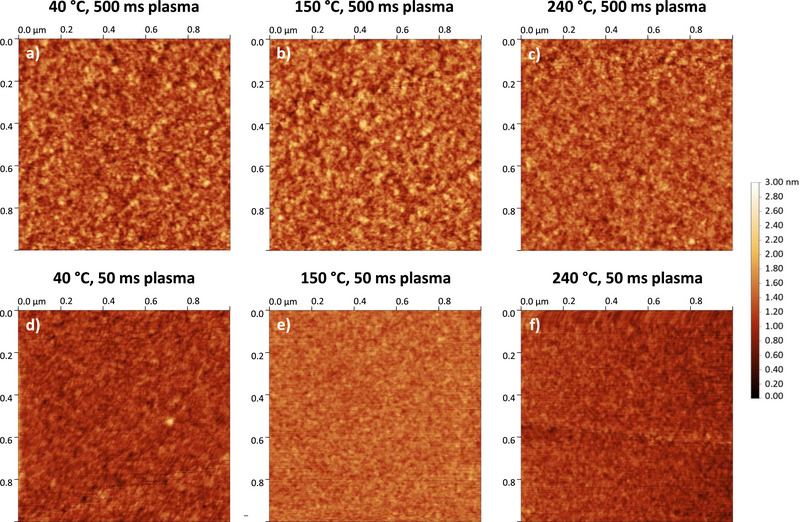
AFM images of GeO_2_ deposited on Si with plasma times of 500 ms (top row) and 50 ms (bottom row), each at temperatures of 40°C, 150°C, and 240°C (from left to right). Each sample was deposited with 500 cycles of the optimized PEALD process.

The surface morphology and roughness of the deposited GeO_2_ film were analyzed by atomic force microscopy (AFM). To assess the effect of process parameters on surface characteristics, films deposited at 40°C, 150°C, and 240°C, each with plasma pulse durations of 500 and 50 ms, were analyzed. The corresponding AFM images are presented in Figure 9, revealing a highly smooth and homogeneous surface with root‐mean‐square roughness (*R_RMS_*) values ranging from 0.30 to 0.19 nm, which is comparable to that of the underlying Si substrate.

As shown in Table 2, there is a slight trend toward decreased roughness at elevated temperatures, likely due to enhanced surface diffusion of precursor molecules during film growth. More notably, the plasma pulse duration significantly affects roughness: samples deposited with 500 ms pulses exhibit grainier surfaces with larger surface features than those processed with 50 ms pulses, which appear smoother. This trend is also reflected in the *R_RMS_* values, which are consistently lower for the short plasma‐pulse samples across all temperatures.

**TABLE 2:** AFM data of GeO_2_ thin films deposited on Si. Each sample was deposited with 500 cycles of the optimized PEALD process.

Plasma [ms]	Temperature [°C]	Thickness [nm]	*R_RMS_* [nm]
500	40	23.7	0.292
500	150	13.8	0.303
500	240	5.2	0.258
50	40	27.7	0.214
50	150	14.9	0.187
50	240	9.1	0.195

This may be explained by reduced interactions between plasma ions and radicals and the film surface when shorter plasma pulses are employed, thereby promoting smoother film growth. This interpretation is consistent with the observation that longer plasma pulses facilitate the formation of sub‐stoichiometric GeO_x_ species. Consequently, shorter plasma durations reduce plasma‐induced surface modifications.

Importantly, no direct correlation between film thickness and surface roughness was observed, demonstrating ALD's ability to produce highly smooth layers regardless of film thickness. Moreover, these findings are promising for the prospective development of 2D germania and its use as a membrane material, where a precise control over the film growth and morphology is essential. Overall, these observations highlight the necessity of carefully evaluating PEALD process parameters to optimize and tailor thin film surface properties.

**FIGURE 10:**
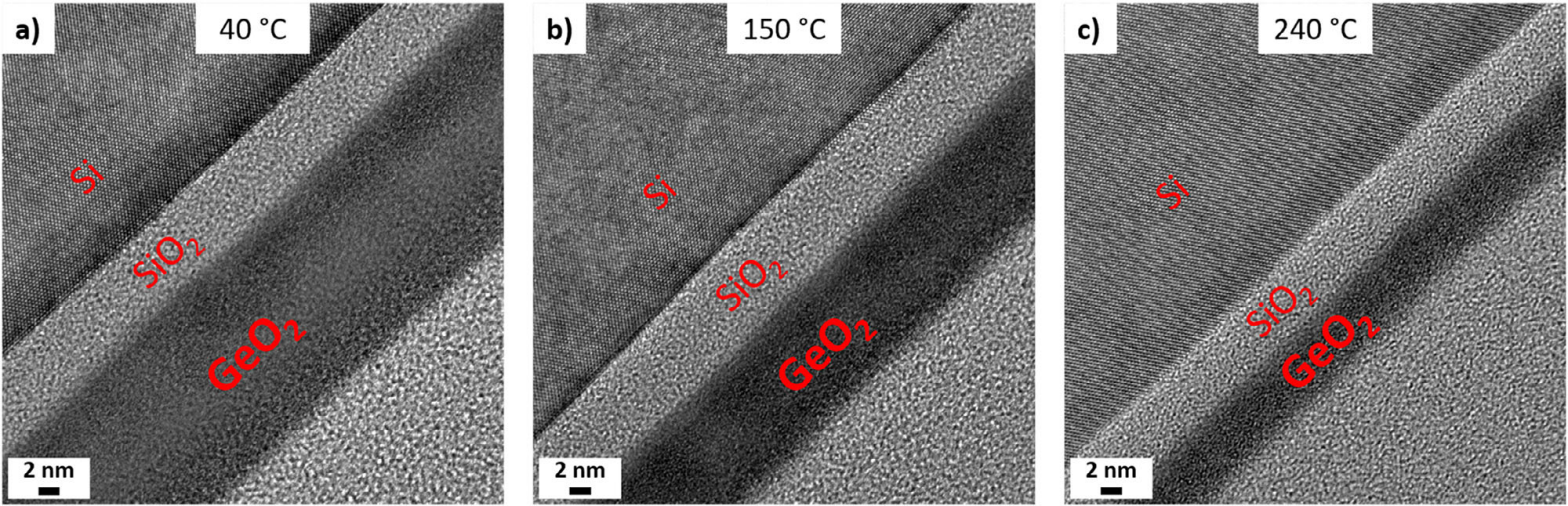
HRTEM images of GeO_2_ thin films deposited with 500 cycles and 50 ms plasma durations at (a) 40°C, (b) 150°C, and (c) 240°C.

The influence of deposition temperature on the film's microstructure and interface was further evaluated by high‐resolution transmission electron microscopy (HRTEM). Cross‐sectional images of samples deposited with 50 ms plasma pulses at 40°C, 150°C, and 240°C reveal a decreasing film thickness with deposition temperature, analogously to the decreasing GPC (Figure 10). Yet all layers are closed and uniform, with a well‐defined interface to the native oxide of the underlying Si substrate. The TEM images confirm an amorphous structure, as evidenced by the absence of crystalline orientation contrast, indicating the absence of grain boundaries.

In the context of dielectric layers, it would be highly interesting to deposit GeO_2_ on etched Si or particularly Ge substrates. This would enable direct evaluation of interface formation and stability on the semiconducting material and thereby provide information on the potential of the grown GeO_2_ as an insulating dielectric layer for Ge‐based high‐power electronics. Such an investigation is, however, exceeding the scope of the present study.

Silica and analogous bilayer structures are commonly fabricated on noble metal substrates, and Au in particular has emerged as a viable option for ALD deposition [45, 92]. As a step toward the fabrication of freestanding bilayer germania by PEALD, the nucleation behavior of the GeO_2_ process was therefore studied by in situ quartz‐crystal microbalance (QCM) analysis using an Au‐coated quartz crystal. The QCM data recorded during the initial PEALD cycles with the optimized sequence and 500 ms plasma exposures at 150°C reveal an initial delay of three cycles, upon which the growth starts and proceeds linearly (Figure S14). This confirms deposition in an ALD‐typical growth mode on the inert Au surface, following a minimal nucleation period and thus demonstrates the possibility of growth on a noble metal, as required for the formation of the bilayer structure [39, 44].

**FIGURE 11:**
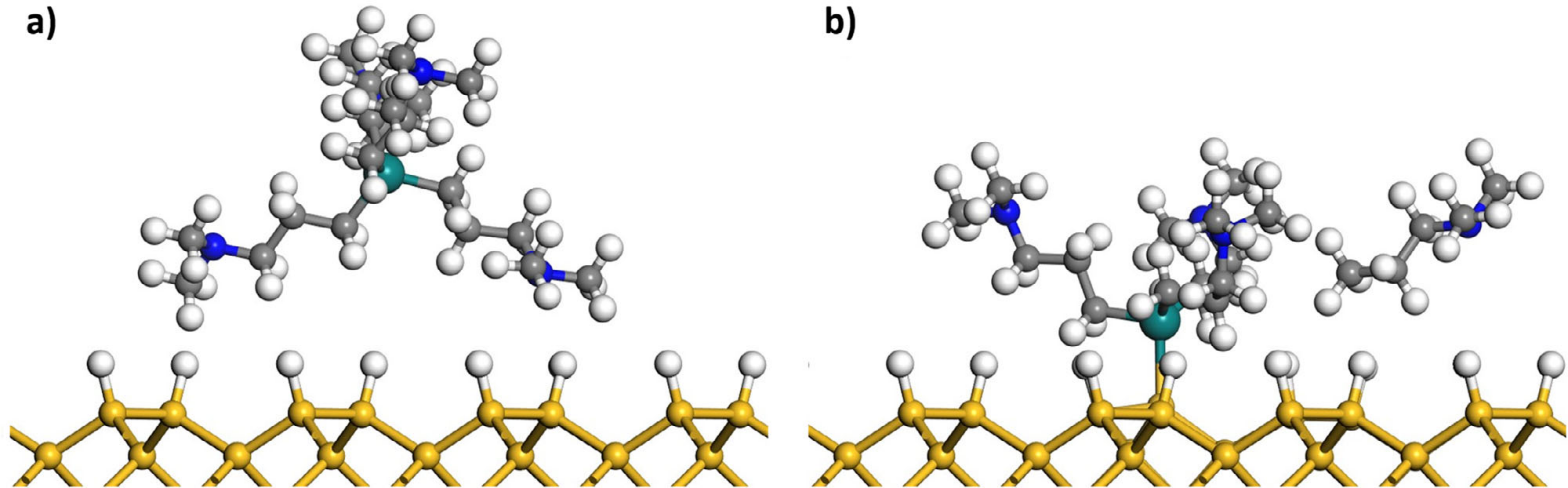
Relaxed DFT structures of (a) physisorption of [Ge(DMP)_4_] on hydrogen‐terminated Si, and (b) chemisorption of [Ge(DMP)_3_] after ligand exchange between precursor and surface, with the detached ligand physisorbed. Grey atoms are C, larger turquoise spheres are Ge, white are H, blue are N, and yellow are Si.

The first step in GeO_x_ deposition, namely the chemisorption of the [Ge(DMP)_4_] precursor, was investigated using DFT calculations on a hydrogen‐terminated Si(100) surface. The intact [Ge(DMP)_4_] weakly physisorbs to the surface, with a computed adsorption energy of −0.53 eV (−0.03 eV without vdW interactions). The significantly larger adsorption energy with vdW corrections confirms that the adsorption is driven primarily by non‐covalent interactions. Both the precursor and the surface show no visible geometric change after physisorption, with the molecule hovering approximately 2.0 Å above the surface, as shown in Figure 11a. Chemisorption was modelled by ligand elimination via surface hydrogen transfer to the DMP ligand. This reaction is exothermic, with an overall energy gain of −1.33 eV (−0.27 eV without vdW interactions), indicating that the precursor's adsorption and ligand elimination are favorable. After the ligand elimination, the remaining [Ge(DMP)_3_] binds to the surface Si with a Ge─Si bond of 2.5 Å (Figure 11b). The detached hydrogenated DMP ligand migrates away and remains weakly adsorbed at the surface. The surface undergoes a slight rearrangement with the binding Si atom migrating 0.2 Å outward from the surface plane. Altogether, these results demonstrate the favorable chemisorption process and are in line with the [Ge(DMP)_3_]^+^ species detected in LIFDI‐MS.

As proof of principle, we attempted to deposit ultrathin GeO_2_ films on Si substrates using eight PEALD cycles at 40°C and 240°C. To obtain stoichiometric GeO_2_, the shorter plasma pulses of 50 ms were employed. Cross‐sectional HRTEM revealed the formation of closed and uniform films with thicknesses of 1.0 nm at 40°C and 0.7 nm at 240°C (Figure S15). Such a rapid and complete nucleation process, achieved with few PEALD cycles, is essential for large‐scale fabrication of the bilayer structure without pinholes or 3D contributions.

## 3 Conclusions

We have established [Ge(DMP)_4_] as a Ge(IV) ALD precursor that meets all prerequisites (high‐purity liquid, volatile, exceptional thermal stability, yet reactive and non‐pyrophoric) and is scalable. SC‐XRD confirms a monomeric structure with monodentate DMP ligands and, to our knowledge, constitutes the first structure of a homoleptic, non‐fluorinated Ge(IV) alkyl complex reported. The coordination environment was additionally evaluated through DFT calculations. Consistent with mass spectrometric analysis, a viable chemisorption route based on the loss of one DMP ligand is proposed.

PEALD with [Ge(DMP)_4_] and oxygen plasma as the co‐reactant produces GeO_2_ and GeO_x_ with adjustable composition. The ALD‐defining self‐limiting deposition is achieved with a linear growth of 0.24 Å per cycle at 150°C, and the reactive precursor allows deposition from 240°C down to 40°C. Notably, the composition of the thin films can be readily controlled by changing the deposition temperature and plasma pulse duration: 50 ms plasma pulses consistently produce GeO_2_ across the temperature range, while 500 ms pulses result in GeO_x_ with x gradually decreasing from approximately 2.1 to about 1.3 as the temperature is increased. Controlling the composition and tuning its optical properties enables the fabrication of GeO_x_ with precisely tailored characteristics to meet device requirements. Future research will also examine how GeO_x_ composition influences the electrical properties of these thin films.

Regardless of composition, the films are pure and uniform, featuring a smooth surface and a well‐defined interface with the substrate, meeting specifications for potential microelectronic and optical applications. In situ QCM and initial downscaling experiments show rapid nucleation on Si and Au surfaces, resulting in closed films with sub‐nm thicknesses. Coupled with the moderate GPC, this offers precise thickness control and provides a promising foundation for exploring 2D germania as a new membrane material through large‐scale PEALD fabrication. Finally, DFT suggests the feasibility of heteroleptic [Ge(DMP)_4_] variants with tuned coordination environments, offering a pathway to developing advanced Ge precursors with customized physico‐chemical properties.

## 4 Experimental Section

### 4.1 General Synthesis Procedure

Handling of air‐ and moisture‐sensitive compounds was carried out in a glovebox (MBraun). All syntheses were conducted in dried and degassed glassware under an argon atmosphere (AirLiquide, 99.995 %) using conventional Schlenk techniques. Solvents were purified and dried using an MBraun‐SPS‐800 purification system. GeCl_4_ (99.9999 %, metal basis) was purchased from Fisher Scientific and used as received without further purification.

### 4.2 Synthesis of Tetrakis‐[3‐(*N*,*N*‐dimethylamino)‐propyl] Germanium(IV) [Ge(DMP)_4_]

[Ge(DMP)_4_] was synthesized in multigram batches by dissolving GeCl_4_ (25.03 g, 60 mmol) in toluene in an ice bath and slowly adding it to a solution of the Grignard reagent [(DMP)MgCl] in THF (freshly prepared according to a literature procedure) [66] at −78°C under rigorous stirring. The resulting slurry was allowed to warm up to room temperature inside the cooling bath under continuous stirring for 12 h. After removal of the solvents, the crude product was extracted with hexane, and insoluble by‐products were filtered off using a cannula fitted with a glass fiber filter. The solvent from the filtrate was removed under reduced pressure, and the crude product was purified by distillation at 100°C and 5 × 10^−3^ mbar, yielding a clear, colorless liquid (16.2 g, 62%). ^1^H NMR (400 MHz, benzene‐*d*_6_, 25°C): δ (ppm) = 2.24 (t, *J*  =  7.1 Hz, 8H, N─CH_2_), 2.15 (s, 24H, N─(CH_3_)_2_), 1.63 (m, 8H, Ge─CH_2_CH_2_) −0.06 (m, 8H, Ge─CH_2_). ^13^C NMR (75 MHz, benzene‐*d_6_*, 25°C): δ (ppm)  =  63.53 (4C, N–CH_2_), 45.69 (8C, N─(CH_3_)_2_), 23.98 (4C, Ge─CH_2_CH_2_), 10.66 (4C, Ge─CH_2_). EA Calc. (%): C 57.57, H 11.60, N 13.43, Ge 17.41; Found (%): C 57.42, H 11.64, N 13.44, Ge 17.32. LIFDI‐MS (*m/z*): [Ge(DMP)_3_]^+^: calc. 332.21, found 332.15.

### 4.3 Precursor Characterization

Storage and sample preparation of [Ge(DMP)_4_] were carried out in an argon‐filled glove box (MBraun LM 100). Deuterated benzene‐*d_6_* purchased from Millipore for NMR experiments was degassed before use and stored over 4 Å molecular sieve. All NMR spectra were recorded on a Bruker Avance III 400 HD instrument and referenced to the internal solvent signal (C_6_D_5_H) and analyzed with the software MestReNova v14.2.1‐27684 from Mestrelab Research S.L. EA of C, H, N, and Ge was conducted by MikroLab Kolbe (Oberhausen, Germany) with a double determination of each element under inert conditions using combustion additives. LIFDI‐MS measurements were performed using a Jeol AccuTOF GCv spectrometer (Freising, Germany), equipped with a LIFDI source from Linden CMS (Weyhe, Germany). TGA and stepped *iso*thermal TGA were conducted on a Hitachi NEXTA STA200 in the temperature range 30°C–550°C. The TG experiments were conducted under a nitrogen atmosphere with standard sample sizes of ∼10–20 mg, a heating rate of 5 K min^−1^ or 20 K min^−1^, and a nitrogen flow rate (AirLiquide, 99.999 %) of 200 mL min^−1^. For the stepped *iso*thermal TG experiment, the temperature was increased stepwise by 10°C, with the heating rate set to 40°C min^−1^ and held constant for 10 min at each step. A linear fit of mass loss determined the respective evaporation rates of each step under consideration of the surface area of the crucible. Single crystals of [Ge(DMP)_4_] were obtained by diluting 0.5 mL of the liquid compound with 1 mL of Et_2_O in a Schlenk flask and storing the mixture in dry ice for several days until colorless crystals formed. Throughout the operation, continuous cooling of the crystals was crucial to prevent melting, which occurred rapidly upon warming above the temperature of dry ice. Under constant dry ice cooling, the solvent was removed using a syringe, and the crystals were covered with X‐Temp oil inside the Schlenk flask. Using an X‐Temp 2 cryostat with a nitrogen stream set to −65°C to ensure cooling, a suitable crystal was selected under a microscope and mounted on an XtaLAB Synergy, Dualflex, HyPix diffractometer. To transfer the crystal from the microscope to the diffractometer, it was submerged in liquid nitrogen, and during data collection, the crystal was maintained in a nitrogen stream at −170°C. Using Olex2 [93], the structure was solved with SHELXT [94] using Intrinsic Phasing and refined with SHELXL [95] using Least‐Squares minimization.

### 4.4 Thin Film Deposition

PEALD depositions were performed in a custom‐built stainless‐steel reactor (modular flow). The reactor chamber consists of a square chamber (20 cm × 20 cm × 20 cm) with a viewport and perpendicular top‐flow (shower‐head) geometry for precursor and gas delivery, and a single‐wafer (2‐inch) grounded substrate holder located between the antennas. A direct electron cyclotron wave resonance (ECWR) O_2_ plasma was generated by a radio frequency generator (13.56 MHz) and an active magnetic flux density of 2.8 mT using a matching network with a plasma power of 100 – 300 W in the pressure range of 10^−2^–10^−3^ mbar [96]. [Ge(DMP)_4_] was filled into stainless‐steel cartridges and heated to 120°C. The plasma power was adjusted to 200 W, whereas oxygen (Air Liquide, 99.995 %) and argon (Air Liquide, 99.995 %) gas flows were adjusted to 15 sccm for all depositions. CZ‐Si(100) p‐type wafers (MicroChemicals) with native oxide (SiO_2_ ∼2 nm) served as substrates for PEALD depositions and process optimization. The wafers were cleaned of dust using pressurized argon. Prior to QCM measurements and depositions on Au/mica substrates, the substrates were exposed to ten 150 ms O_2_ plasma pulses, separated by 850 ms of vacuum at the desired deposition temperature. Afterward, the PEALD process was performed with the optimized pulse/purge scheme and plasma durations of 500 ms or 50 ms (Figure 5, top).

### 4.5 Thin Film Characterization

In situ QCM experiments were conducted using Au‐coated AT‐cut quartz crystals (*f_res_* = 6 MHz). The crystal holder was custom‐built and connected to an SO‐100 Oscillator and an SQM‐160 thin film deposition monitor (JCM, Inficon). The thickness and density of GeO_x_ films were determined by X‐ray reflectometry (XRR; Bruker D8 Discover XRD) with Cu‐K_α_ radiation (*λ* = 1.5418 Å) in Θ–2Θ locked coupled mode. 2Θ was increased from 0.1° to 3° with a step size of 0.01° and a scan speed of 1 s. The composition of selected thin films on Si(100) substrates was determined using two ion‐beam analytical methods at the 4 MV tandem accelerator facility RUBION at Ruhr University Bochum, with an incident tilt angle of 7°. RBS measurements were performed with a ^4^He^+^ ion beam of 2.0 MeV (intensity 40 – 50 nA), allowing a good quantification of the higher Z elements (here Ge). For the determination of O content and with high sensitivity to C, and N contaminations, nuclear reaction analysis (NRA) was performed using an ion beam of 1.0 MeV deuterons. For RBS measurements, a silicon detector was used for the backscattered He‐nuclei at an angle of 160°, while for NRA, the detection angle for the emitted protons was 135°. The SIMNRA program [97] was employed for RBS and NRA raw data processing and analysis.

XPS studies were carried out on a PHI 5600 instrument for the sample deposited with 500 ms plasma exposure at 150°C and on a PHI 5000 VersaProbe II instrument for all other samples, utilizing Al‐K_α_ photon radiation (1486.6 eV). GeO_x_ thin‐film samples were analyzed using a combination of survey and core‐level scans to identify peaks of interest. Step widths were adjusted to 0.5 eV for each survey scan and 0.05 eV for the core level scans. Hereby, the pass energies were adjusted to 187.5 and 23.5 eV (29.5 eV for the PHI 5600 instrument), respectively. All binding energies of Ge 3d and O 1s were referenced to the signal of adventitious carbon species (284.8 eV) [90]. The analysis chamber pressure was maintained at <10^−7^ mbar. Recordings were taken of the as‐introduced surface followed by scans after an Ar^+^‐sputter step (2 min, 3 kV, 2 × 2). The sputter step significantly altered the Ge oxidation states; therefore, chemical species analysis was limited to the as‐introduced surfaces. The deconvolution analysis was performed using Shirley background subtraction and Gaussian functions in Casa XPS [98]. The refractive index of GeO_x_ films was measured by spectroscopic ellipsometry using an M‐2000 V ellipsometer from J.A. Woollam, Inc. (370–1000 nm). Total reflectance UV/vis spectra of GeO_x_ thin films were measured using a Shimadzu UV‐3600i Plus spectrophotometer with a bare Si(100) substrate with native oxide as a reference. AFM measurements of selected samples were conducted using a Dimension Icon (Bruker) in tapping mode, with a TESPA‐V2 cantilever, inside an antivibration box. A line‐scan rate of 1 Hz with a 1024 × 1024 pixel resolution was used for measurements, with a scan range of 1 µm × 1 µm. Recorded images were processed with Nanoscope Analysis 1.8 (Bruker) software, applying line centering by tilt and bow correction where necessary before calculating the roughness parameters. TEM was performed on a Tecnai F30 (FEI) operated at 300 kV, equipped with a high‐angle annular dark‐field (HAADF) detector for scanning imaging and an energy‐dispersive X‐ray spectrometer (EDXS) (TEAM Octane T Optima EDS windowless, Edax/Ametek). Cross‐sectional specimens were prepared for TEM using a dual‐beam focused ion beam (FIB) microscope (Helios 5 CX, TFS) operated at 30 kV. As a final specimen preparation step 5 kV ion beam cleaning was applied to the TEM sample to reduce damage by the FIB process. Cross‐sectional images of the specimens were obtained using bright‐field, high‐resolution TEM.

### 4.6 Density Functional Theory Calculations

Non‐periodic DFT calculations were performed using the TURBOMOLE software suite [99, 100]. The hybrid functional PBE0 was used as the exchange‐correlation functional, mixing the 0.25 exact Hartree‐Fock exchange with 0.75 PBE exchange and full PBE correlation, with the m3 integration grid [101, 102]. The polarized triple zeta valence basis set def2‐TZVP was used for all atoms [103, 104]. Geometry optimizations were performed using internal redundant coordinates. The convergence criteria for the electronic SCF cycles were set to 10^−6^ Ha and the convergence criteria for the geometry optimization were set to the energy gradient less than 10^−3^. [Ge(DMP)_4_] was in a closed‐shell singlet configuration, while [Ge(DMP)_3_] and the isolated DMP ligand were in doublet states. The ligand elimination energy was calculated using Equation (2).

(2) $$\begin{array}{c} \Delta_{elim}E=E_{\mathrm{Ge}{\left(\mathrm{DMP}\right)}_{3}}+E_{\mathrm{DMP}}\;-E_{\mathrm{Ge}{\left(\mathrm{DMP}\right)}_{4}} \end{array}$$

The Vienna Ab initio Simulation Package (VASP) was used for periodic DFT calculations [105, 106, 107]. The generalized gradient approximation exchange‐correlation functional form Perdew, Burke, and Ernzerhof (PBE), was used together with Grimme's D3 dispersion correction [101, 108]. Projector augmented wave potentials were used to treat core electrons, and plane waves with a cutoff energy of 550 eV were used for valence electrons [109]. The valence electron configurations used were 2s^2^2p^2^ for C, 3d^10^4s^2^4p^2^ for Ge, 1s^1^ for H, 2s^2^2p^3^ for N, 3s^2^3p^2^ for Si, and 2s^2^2p^4^ for N. All calculations had a Gaussian smearing with a width of 0.1. Oxygen vacancies were calculated using spin‐polarized calculations with an unrestricted spin, while adsorption assumed non‐spin polarization. The convergence criteria were 10^−4^ eV for the electronic cycles and 0.02 eV Å^−1^ for the ionic relaxation.

A 2 × 2 × 2 supercell of rutile GeO_2_, with composition Ge_16_O_32_ and the DFT relaxed cell parameters a = b = 9.03 Å, c = 5.82 Å, and α = β = γ = 90°. For the oxygen vacancy, one oxygen atom was removed from the cell structure. A 3 × 3 × 4 Monkhorst‐Pack k‐point grid was used for bulk calculations [110]. For the adsorption calculations, a surface slab of Si was cut along the (100) direction of diamond‐structured bulk Si, yielding 8 Si layers. The slab was padded with 15 Å of vacuum and a 5 × 5 × 2 supercell for the precursor adsorption calculations. The top and bottom of the slab were passivated by one H atom per surface Si atom and relaxed into a (2 × 1) surface reconstruction.

## Conflicts of Interest

The authors declare no conflicts of interest.

## Supporting information



The authors have cited additional references within the Supporting Information [57, 59, 64, 111, 112, 113, 114].
Deposition Number 2485525 contains the supplementary crystallographic data for [Ge(DMP)_4_]. This data is provided free of charge by the joint Cambridge Crystallographic Data Centre and Fachinformationszentrum Karlsruhe Access Structures service.
**Supporting File**: smll72769‐sup‐0001‐SuppMat.pdf.

## Data Availability

The data that support the findings of this study are available from the corresponding author upon reasonable request.

## References

[1] M. Labed , H. J. Jeon , J. H. Park , S. J. Pearton , and Y. S. Rim , “Rutile Germanium Dioxide: An Emerging Ultrawide Bandgap Semiconductor for Power Device Applications—A Review,” Materials Today 83 (2025): 513–537, 10.1016/j.mattod.2025.01.012.

[2] C. H. Lee , T. Tabata , T. Nishimura , K. Nagashio , K. Kita , and A. Toriumi , “Ge/GeO_2_ Interface Control with High‐Pressure Oxidation for Improving Electrical Characteristics,” Applied Physics Express 2 (2009): 71404, 10.1143/APEX.2.071404.

[3] S. Chae , J. Lee , K. A. Mengle , J. T. Heron , and E. Kioupakis , “Rutile GeO_2_: An Ultrawide‐band‐gap Semiconductor with Ambipolar Doping,” Applied Physics Letters 114 (2019): 102104.

[4] M. Perego , G. Scarel , M. Fanciulli , I. L. Fedushkin , and A. A. Skatova , “Fabrication of GeO_2_ Layers Using a Divalent Ge Precursor,” Applied Physics Letters 90 (2007): 162115.

[5] A. Delabie , A. Alian , F. Bellenger , et al., “H_2_O‐ and O_3_‐Based Atomic Layer Deposition of High‐κ Dielectric Films on GeO_2_ Passivation Layers,” Journal of the Electrochemical Society 156 (2009): G163, 10.1149/1.3200902.

[6] A. Delabie , F. Bellenger , M. Houssa , et al., “Effective Electrical Passivation of Ge(100) for High‐k Gate Dielectric Layers Using Germanium Oxide,” Applied Physics Letters 91 (2007): 082904.

[7] K. Bushick , K. A. Mengle , S. Chae , and E. Kioupakis , “Electron and Hole Mobility of Rutile GeO_2_ from First Principles: An Ultrawide‐bandgap Semiconductor for Power Electronics,” Applied Physics Letters 117 (2020): 182104.

[8] M. U. Dzhanklych , V. G. Dyskin , and Z. S. Settarova , “Antireflection Coatings for Solar Elements Based on a Composition of Ge and GeO_2_ ,” Applied Solar Energy 46 (2010): 130–132, 10.3103/S0003701X1002012X.

[9] J. A. Yousif , S. Alptekin , and A. Ramizy , “Preparation and Characterization of Germanium Dioxide Nanostructure for Gas Sensor Application: Effect of Laser Parameters,” Digest Journal of Nanomaterials and Biostructures 18 (2023): 1139–1146, 10.15251/DJNB.2023.183.1139.

[10] A. R. Phani , D. Di Claudio , M. Passacantando , and S. Santucci , “GeO_2_‐based High k Dielectric Material Synthesized by Sol–Gel Process,” Journal of Non‐Crystalline Solids 353 (2007): 692–696, 10.1016/j.jnoncrysol.2006.10.040.

[11] M. G. Zeariya , S. K. M. El‐Shennawy , A. Kassar , et al., “Chitosan Thin Films Developed with Germanium Oxide for Wound Healing Applications: Cell Viability, Wettability, and Antibacterial Activity,” Materials Chemistry and Physics 328 (2024): 129968, 10.1016/j.matchemphys.2024.129968.

[12] N. P. S. Chauhan , M. Gholipourmalekabadi , and M. Mozafari , “Fabrication of Newly Developed Pectin –GeO_2_ Nanocomposite Using Extreme Biomimetics Route and its Antibacterial Activities,” Journal of Macromolecular Science, Part A 54 (2017): 655.

[13] D. E.‐S. Ellakwa , A. S. Abu‐Khadra , and T. E. Ellakwa , “Insight into Bioactive Glass and Bio‐ceramics Uses: Unveiling Recent Advances for Biomedical Application,” Discover Materials 5 (2025): 78.

[14] T. M. Tiama , M. A. Ibrahim , M. H. Sharaf , and A. F. Mabied , “Effect of Germanium Oxide on the Structural Aspects and Bioactivity of Bioactive Silicate Glass,” Scientific Reports 13 (2023): 9582, 10.1038/s41598-023-36649-5. 37311789 PMC10264357

[15] K. Prabhakaran and T. Ogino , “Oxidation of Ge(100) and Ge(111) Surfaces: An UPS and XPS Study,” Surface Science 325 (1995): 263–271, 10.1016/0039-6028(94)00746-2.

[16] R. J. Theeuwes , W. M. M. Kessels , and B. Macco , “Surface Passivation Approaches for Silicon, Germanium, and III–V Semiconductors,” Journal of Vacuum Science and Technology, A (2024): 42.

[17] L. Zhang , H. Li , Y. Guo , et al., “Selective Passivation of GeO_2_/Ge Interface Defects in Atomic Layer Deposited High‐k MOS Structures,” ACS Applied Materials and Interfaces 7 (2015): 20499–20506, 10.1021/acsami.5b06087. 26334784

[18] J.‐S. Jung , D.‐H. Kim , J.‐H. Shin , and J.‐G. Kang , “Atomic Layer Deposition of GeO_2_ Thin Films on Si(100) using Ge( N , N ′‐R,R‐en)( NMe_2_)_2_ (Where R = Isopropyl and t ‐Butyl) Precursors,” Bulletin of the Korean Chemical Society 36 (2015): 1953–1954, 10.1002/bkcs.10400.

[19] G. A. Lyashchenko , “Electrical Properties of Defective Germanium Monoxide Films,” Soviet Physics Journal 13 (1970): 1220–1223, 10.1007/BF01100558.

[20] G. Pérez , A. M. Bernal‐Oliva , E. Márquez , et al., “Optical and Structural Characterization of Single and Multilayer Germanium/Silicon Monoxide Systems,” Thin Solid Films 485 (2005): 274.

[21] P. J. Wolf , T. M. Christensen , N. G. Coit , and R. W. Swinford , “Thin Film Properties of Germanium Oxide Synthesized by Pulsed Laser Sputtering in Vacuum and Oxygen Environments,” Journal of Vacuum Science & Technology A: Vacuum, Surfaces, and Films 11 (1993): 2725–2732, 10.1116/1.578633.

[22] E. Gorokhov , K. Astankova , and A. Komonov , “GeO_2_ Films with Ge‐Nanoclusters in Layered Compositions: Structural Modifications with Laser Pulses,” in Laser Pulses. Theory, Technology, and Applications, (Eds:. I. Peshko ), (IntechOpen, 2012), ISBN 978‐953‐51‐0796‐5.

[23] K. N. Astankova , V. A. Volodin , and I. A. Azarov , “Structure of Germanium Monoxide Thin Films,” Semiconductor 54 (2020): 1555.

[24] J. Y. Lee , Y. Kim , M.‐H. Kim , et al., “Ni/GeO_x_/p^+^ Si Resistive‐switching Random‐access Memory with Full Si Processing Compatibility and its Characterization and Modeling,” Vacuum 161 (2019): 63–70, 10.1016/j.vacuum.2018.12.020.

[25] K. Udaya Mohanan , S. Cho , and B.‐G. Park , “Medium‐Temperature‐Oxidized GeO_x_ Resistive‐Switching Random‐Access Memory and Its Applicability in Processing‐in‐Memory Computing,” Nanoscale Research Letters 17 (2022): 63, 10.1186/s11671-022-03701-8. 35789299 PMC9256894

[26] A. Prakash , S. Maikap , S. Z. Rahaman , S. Majumdar , S. Manna , and S. K. Ray , “Resistive Switching Memory Characteristics of Ge/GeO_x_ Nanowires and Evidence of Oxygen Ion Migration,” Nanoscale Research Letters 8 (2013): 220, 10.1186/1556-276X-8-220. 23657016 PMC3686581

[27] B. Son , Y. Lin , K. H. Lee , Y. Wang , S. Wu , and C. S. Tan , “High Speed and Ultra‐low Dark Current Ge Vertical *p–i–n* Photodetectors on an Oxygen‐annealed Ge‐on‐insulator Platform With GeO_x_ Surface Passivation,” Optics Express 28 (2020): 23978, 10.1364/OE.398199. 32752385

[28] N. Hohn , X. Wang , M. A. Giebel , et al., “Mesoporous GeO_x_/Ge/C as a Highly Reversible Anode Material with High Specific Capacity for Lithium‐Ion Batteries,” ACS Applied Materials and Interfaces 12 (2020): 47002–47009, 10.1021/acsami.0c13560. 32955236

[29] C. Liu , Y. Zhao , R. Yi , et al., “Enhanced Electrochemical Performance by GeO_x_‐coated MXene Nanosheet Anode in Lithium‐ion Batteries,” Electrochimica Acta 358 (2020): 136923, 10.1016/j.electacta.2020.136923.

[30] X.‐L. Wang , W.‐Q. Han , H. Chen , et al., “Amorphous Hierarchical Porous GeO_x_ as High‐Capacity Anodes for Li Ion Batteries With Very Long Cycling Life,” Journal of the American Chemical Society 133 (2011): 20692–20695, 10.1021/ja208880f. 22141466

[31] K. Shen , N. Lin , T. Xu , Y. Han , and Y. Qian , “Amorphous Mesoporous GeO_x_ Anode for Na‐ion Batteries With High Capacity and Long Lifespan,” Royal Society Open Science 5 (2018): 171477, 10.1098/rsos.171477. 29410850 PMC5792927

[32] T. Kajita and T. Itoh , “Ether‐based Solvents Significantly Improved Electrochemical Performance for Na‐ion Batteries with Amorphous GeO_x_ Anodes,” Physical Chemistry Chemical Physics 19 (2017): 1003–1009, 10.1039/C6CP06354C. 27790663

[33] N. R. Murphy , J. T. Grant , L. Sun , et al., “Correlation Between Optical Properties and Chemical Composition of Sputter‐deposited Germanium Oxide (GeO_x_) Films,” Optical Materials 36 (2014): 1177–1182, 10.1016/j.optmat.2014.02.023.

[34] J. Cai , X. Han , X. Wang , and X. Meng , “Atomic Layer Deposition of Two‐Dimensional Layered Materials: Processes, Growth Mechanisms, and Characteristics,” Matter 2 (2020): 587–630, 10.1016/j.matt.2019.12.026.

[35] D. Löffler , J. J. Uhlrich , M. Baron , et al., “Growth and Structure of Crystalline Silica Sheet on Ru(0001),” Physical Review Letters 105 (2010): 146104, 10.1103/PhysRevLett.105.146104. 21230849

[36] L. Lichtenstein , C. Büchner , B. Yang , et al., “The Atomic Structure of a Metal‐Supported Vitreous Thin Silica Film,” Angewandte Chemie International Edition 51 (2012): 404–407, 10.1002/anie.201107097. 22114049

[37] E. I. Altman and U. D. Schwarz , “Structural and Electronic Heterogeneity of Two Dimensional Amorphous Silica Layers,” Advanced Materials Interfaces 1 (2014): 1400108.

[38] C. Büchner and M. Heyde , “Two‐Dimensional Silica Opens New Perspectives,” Progress in Surface Science 92 (2017): 341.

[39] A. Malashevich , S. Ismail‐Beigi , and E. I. Altman , “Directing the Structure of 2D Silica and Silicates,” The Journal of Physical Chemistry C 120 (2016): 26770–26781, 10.1021/acs.jpcc.6b07008.

[40] A. K. Singh , B. C. Revard , R. Ramanathan , M. Ashton , F. Tavazza , and R. G. Hennig , “Genetic Algorithm Prediction of Two‐dimensional Group‐IV Dioxides for Dielectrics,” Physical Review B 95 (2017): 155426.

[41] F. Zhao , Y. Feng , and W. Feng , “Germanium‐based Monoelemental and Binary 2D Materials: Theoretical and Experimental Investigations and Promising Applications,” InfoMat 4 (2022): 12365.

[42] A. L. Lewandowski , S. Tosoni , L. Gura , et al., “From Crystalline to Amorphous Germania Bilayer Films at the Atomic Scale: Preparation and Characterization,” Angewandte Chemie International Edition 58 (2019): 10903–10908, 10.1002/anie.201903922. 31050096 PMC6771709

[43] A. L. Lewandowski , P. Schlexer , S. Tosoni , et al., “Determination of Silica and Germania Film Network Structures on Ru(0001) at the Atomic Scale,” The Journal of Physical Chemistry C 123 (2019): 7889–7897, 10.1021/acs.jpcc.8b07110.

[44] A. L. Lewandowski , S. Tosoni , L. Gura , et al., “Growth and Atomic‐Scale Characterization of Ultrathin Silica and Germania Films: The Crucial Role of the Metal Support,” Chemistry—A European Journal 27 (2021): 1870–1885, 10.1002/chem.202001806. 33118653 PMC7898484

[45] D. Naberezhnyi , L. Mai , N. Doudin , et al., “Molecular Permeation in Freestanding Bilayer Silica,” Nano Letters 22 (2022): 1287–1293, 10.1021/acs.nanolett.1c04535. 35044780

[46] P. Dementyev , N. Khayya , D. Zanders , I. Ennen , A. Devi , and E. I. Altman , “Size and Shape Exclusion in 2D Silicon Dioxide Membranes,” Small 19 (2023): 2205602. 10.1002/smll.202205602 36521931

[47] E. I. Altman and P. Dementyev , “Atomic Layer Deposition Brings Applications of Two‐Dimensional Silica to the Fore,” Catalysis Letters 154 (2024): 1359–1374, 10.1007/s10562-023-04435-7.

[48] D. Jiang , V. R. Cooper , and S. Dai , “Porous Graphene as the Ultimate Membrane for Gas Separation,” Nano Letters 9 (2009): 4019–4024, 10.1021/nl9021946. 19995080

[49] S. Homaeigohar and M. Elbahri , “Graphene Membranes for Water Desalination,” NPG Asia Materials 9 (2017): e427–e427, 10.1038/am.2017.135.

[50] S. M. George , “Atomic Layer Deposition: An Overview,” Chemical Reviews 110 (2010): 111–131, 10.1021/cr900056b. 19947596

[51] A. Pakkala and M. Putkonen in Handbook of Deposition Technologies for Films and Coatings: Science, Applications and Technology, ed. P. M. Martin (Elsevier, 2010), 364–391.

[52] M. Leskelä and M. Ritala , “Atomic Layer Deposition Chemistry: Recent Developments and Future Challenges,” Angewandte Chemie International Edition 42 (2003): 5548–5554, 10.1002/anie.200301652. 14639717

[53] L. Hu , W. Qi , and Y. Li , “Coating Strategies for Atomic Layer Deposition,” Nanotechnology Reviews 6 (2017): 527–547, 10.1515/ntrev-2017-0149.

[54] F. Rahman and J. C. Runyon , “Atomic Layer Processes for Material Growth and Etching—A Review,” IEEE Transactions on Semiconductor Manufacturing 34 (2021): 500–512, 10.1109/TSM.2021.3112502.

[55] A. Devi , “‘Old Chemistries’ for New Applications: Perspectives for Development of Precursors for MOCVD and ALD Applications,” Coordination Chemistry Reviews 257 (2013): 3332–3384, 10.1016/j.ccr.2013.07.025.

[56] T. Hatanpää , M. Ritala , and M. Leskelä , “Precursors as Enablers of ALD Technology: Contributions from University of Helsinki,” Coordination Chemistry Reviews 257 (2013): 3297–3322, 10.1016/j.ccr.2013.07.002.

[57] C. M. Yoon , I.‐K. Oh , Y. Lee , et al., “Water‐Erasable Memory Device for Security Applications Prepared by the Atomic Layer Deposition of GeO_2_ ,” Chemistry of Materials 30 (2018): 830–840, 10.1021/acs.chemmater.7b04371.

[58] J. Antoja‐Lleonart , S. Zhou , K. de Hond , et al., “Atomic Layer Deposition of SiO_2_–GeO_2_ Multilayers,” Applied Physics Letters 117 (2020): 041601.

[59] H. Choi , C. Park , S. K. Lee , et al., “New Heteroleptic Germanium Precursors for GeO_2_ Thin Films by Atomic Layer Deposition,” ACS Omega 8 (2023): 43759–43770, 10.1021/acsomega.3c05657. 38027341 PMC10666237

[60] A. Hultqvist , J. Keller , N. M. Martin , F. Larsson , and T. Törndahl , “Sn_1– x_ Ge_x_ O_y_ and Zn_1–x_ Ge_x_ O_y_ by Atomic Layer Deposition─Growth Dynamics, Film Properties, and Compositional Tuning for Charge Selective Transport in (Ag,Cu)(In,Ga)Se_2_ Solar Cells,” ACS Applied Energy Materials 6 (2023): 9824–9836, 10.1021/acsaem.3c00960.

[61] D. Shin , H. Park , S. Y. Kim , and D.‐H. Ko , “Study on Temperature‐dependent Growth Characteristics of Germanium Oxide Film by Plasma‐enhanced Atomic Layer Deposition,” Thin Solid Films 775 (2023): 139851, 10.1016/j.tsf.2023.139851.

[62] M. Kanematsu , S. Shibayama , M. Sakashita , W. Takeuchi , O. Nakatsuka , and S. Zaima , “Effect of GeO_2_ Deposition Temperature in Atomic Layer Deposition on Electrical Properties of Ge Gate Stack,” Japanese Journal of Applied Physics 55 (2016): 08PC05, 10.7567/JJAP.55.08PC05.

[63] L. Mai , N. Boysen , D. Zanders , et al., “Potential Precursor Alternatives to the Pyrophoric Trimethylaluminium for the Atomic Layer Deposition of Aluminium Oxide,” Chemistry—A European Journal 25 (2019): 7489–7500, 10.1002/chem.201900475. 30870572

[64] L. Mai , D. Zanders , E. Subaşı , et al., “Low‐Temperature Plasma‐Enhanced Atomic Layer Deposition of Tin(IV) Oxide From a Functionalized Alkyl Precursor: Fabrication and Evaluation of SnO_2_‐Based Thin‐Film Transistor Devices,” ACS Applied Materials and Interfaces 11 (2019): 3169–3180, 10.1021/acsami.8b16443. 30624887

[65] L. Mai , F. Mitschker , C. Bock , et al., “From Precursor Chemistry to Gas Sensors: Plasma‐Enhanced Atomic Layer Deposition Process Engineering for Zinc Oxide Layers From a Nonpyrophoric Zinc Precursor for Gas Barrier and Sensor Applications,” Small 16 (2020): 1907506. 10.1002/smll.201907506 32346997

[66] F. Preischel , K. Rönnby , L. Mai , et al., “Near Room‐Temperature Atomic Layer Deposition of Magnesium Oxide Using Bis‐3‐(*N*,*N*‐dimethylamino)propyl Magnesium(II) and Water,” Journal of the American Chemical Society 147 (2025): 31764–31778, 10.1021/jacs.5c08514. 40851270

[67] M. Lapteva , V. Beladiya , S. Riese , et al., “Influence of Temperature and Plasma Parameters on the Properties of PEALD HfO_2_ ,” Optical Materials Express 11 (2021): 1918, 10.1364/OME.422156.

[68] F. Preischel , D. Zanders , T. Berning , et al., “Interplay of Precursor and Plasma for the Deposition of HfO_2_ via PEALD: Film Growth and Dielectric Properties,” Adv Mater Inter 10 (2023): 2300244.

[69] D. L. Tabern , W. R. Orndorff , and L. M. Dennis , “GERMANIUM. XII. Tetra‐Alkyl and Tetra‐Aryl Compounds of Germanium. Germanium Tetra‐Ethoxyl 1,” Journal of the American Chemical Society 47 (1925): 2039–2044, 10.1021/ja01684a038.

[70] E. H. Brooks , F. Glockling , J. Ward , and W. A. G. Graham , in Inorganic Syntheses, Vol. 12, ed. R. W. Parry , (Wiley, 1970): 58–59.

[71] A. Zickgraf , M. Beuter , U. Kolb , et al., “Nucleophilic attack Within Ge, Sn and Pb complexes containing Me2N(CH2)3—as a potential intramolecular donor ligand,” Inorganica Chimica Acta 275–276 (1998): 203–214, 10.1016/S0020-1693(98)00071-1.

[72] L. Mai , M. Gebhard , T. de Los Arcos , et al., “Unearthing 3‐(Dimethylamino)propylaluminium(III) Complexes as Novel Atomic Layer Deposition (ALD) Precursors for Al_2_O_3_: Synthesis, Characterization and ALD Process Development,” Chemistry 23 (2017): 10768. 28665519 10.1002/chem.201702939

[73] H. K. Hofstee , J. Boersma , J. D. van der Meulen , and G. van der Kerk , “Synthesis and Coordination Properties of ω‐functionally‐substituted Dialkylzinc Compounds,” Journal of Organometallic Chemistry 153 (1978): 245–252, 10.1016/S0022-328X(00)92046-1.

[74] L. Yang , D. R. Powell , and R. P. Houser , “Structural Variation in Copper(i) Complexes with Pyridylmethylamide Ligands: Structural Analysis with a New Four‐Coordinate Geometry Index, τ 4,” Dalton Transactions 36 (2007): 955–964, 10.1039/B617136B. 17308676

[75] A. Okuniewski , D. Rosiak , J. Chojnacki , and B. Becker , “Coordination Polymers and Molecular Structures Among Complexes of Mercury(II) Halides with Selected 1‐benzoylthioureas,” Polyhedron 90 (2015): 47–57, 10.1016/j.poly.2015.01.035.

[76] M. Wolf , A. Falk , M. Flock , A. Torvisco , and F. Uhlig , “Selective Synthesis of Tetraarylgermanes and Triarylgermanium Halides,” Journal of Organometallic Chemistry 851 (2017): 143–149, 10.1016/j.jorganchem.2017.09.027.

[77] S. Pelzer , B. Neumann , H.‐G. Stammler , N. Ignat'ev , R. Eujen , and B. Hoge , “Synthesis and Characterization of Tetrakis(pentafluoroethyl)germane,” Synthesis 49 (2017): 2389.

[78] C. Winkler , “Mittheilungen über Das Germanium,” Journal für Praktische Chemie 36 (1887): 177–209, 10.1002/prac.18870360119.

[79] L. M. Dennis , “Germanium Zusammenfassung der Untersuchungen im Department of Chemistry, Cornell University, 1921–1927,” Zeitschrift für anorganische und allgemeine Chemie 174 (1928): 97–141, 10.1002/zaac.19281740114.

[80] L. M. Dennis and F. E. Hance , “Germanium. IX. Germanium Tetra‐Ethyl. Preparation and Purification of Zinc Diethyl. Analysis by Combustion of a Liquid Containing Carbon and Hydrogen 1,” Journal of the American Chemical Society 47 (1925): 370–379, 10.1021/ja01679a013.

[81] P. Kaur , L. Mai , A. Muriqi , et al., “Cover Feature: Rational Development of Guanidinate and Amidinate Based Cerium and Ytterbium Complexes as Atomic Layer Deposition Precursors: Synthesis, Modeling, and Application (Chem. Eur. J. 15/2021),” Chemistry—A European Journal 27 (2021): 4758, 10.1002/chem.202005268. PMC7986905 33470473

[82] M. Wilken , A. Muriqi , A. Krusenbaum , M. Nolan , and A. Devi , “Targeting Manganese Amidinate and Ss‐Ketoiminate Complexes as Precursors for Mn‐Based Thin Film Deposition,” Chemistry (Weinheim An Der Bergstrasse, Germany) 30 (2024): 202401275. 10.1002/chem.202401275 38656605

[83] T. J. Knisley , L. C. Kalutarage , and C. H. Winter , “Precursors and Chemistry for the Atomic Layer Deposition of Metallic First Row Transition Metal Films,” Coordination Chemistry Reviews 257 (2013): 3222–3231, 10.1016/j.ccr.2013.03.019.

[84] S. E. Koponen , P. G. Gordon , and S. T. Barry , “Principles of Precursor Design for Vapor Deposition Methods,” Polyhedron 108 (2016): 59–66, 10.1016/j.poly.2015.08.024.

[85] I. Langmuir , “The Vapor Pressure of Metallic Tungsten,” Physical Review 2 (1913): 329–342, 10.1103/PhysRev.2.329.

[86] G. V. Kunte , S. A. Shivashankar , and A. M. Umarji , “Thermogravimetric Evaluation of the Suitability of Precursors for MOCVD,” Measurement Science and Technology 19 (2008): 025704, 10.1088/0957-0233/19/2/025704.

[87] P. W. Atkins and J. de Paula , Atkins' Physical Chemistry, Ed:. (W.H. Freeman, 2006), ISBN 978‐0716787594.

[88] K. Prabhakaran , F. Maeda , Y. Watanabe , and T. Ogino , “Distinctly Different Thermal Decomposition Pathways of Ultrathin Oxide Layer on Ge and Si Surfaces,” Applied Physics Letters 76 (2000): 2244–2246, 10.1063/1.126309.

[89] K. Prabhakaran , F. Maeda , Y. Watanabe , and T. Ogino , “Thermal Decomposition Pathway of Ge and Si Oxides: Observation of a Distinct Difference,” Thin Solid Films 369 (2000): 289–292, 10.1016/S0040-6090(00)00881-6.

[90] T. L. Barr and S. Seal , “Nature of the Use of Adventitious Carbon as a Binding Energy Standard,” Journal of Vacuum Science & Technology A: Vacuum, Surfaces, and Films 13 (1995): 1239–1246, 10.1116/1.579868.

[91] S. McDonnell , Spectroscopic Characterisation of High Dielectric Constant Materials on Semiconducting Surfaces, (PhD Thesis Dublin City University, 2009).

[92] G. S. Hutchings , X. Shen , C. Zhou , et al., “Scalable Production of Single 2D van der Waals Layers Through Atomic Layer Deposition: Bilayer Silica on Metal Foils and Films,” 2D Materials 9 (2022): 21003.

[93] O. V. Dolomanov , L. J. Bourhis , R. J. Gildea , J. A. K. Howard , and H. Puschmann , “OLEX2: A Complete Structure Solution, Refinement and Analysis Program,” Journal of Applied Crystallography 42 (2009): 339–341, 10.1107/S0021889808042726.

[94] G. M. Sheldrick , “SHELXT—integrated Space‐group and Crystal‐structure Determination,” Acta Crystallographica Section A Foundations and Advances 71 (2015): 3–8, 10.1107/S2053273314026370. 25537383 PMC4283466

[95] G. M. Sheldrick , “Crystal Structure Refinement With SHELXL,” Acta Crystallographica Section C Structural Chemistry 71 (2015): 3–8, 10.1107/S2053229614024218. 25567568 PMC4294323

[96] M. Gebhard , L. Mai , L. Banko , et al., “PEALD of SiO_2_ and Al_2_O_3_ Thin Films on Polypropylene: Investigations of the Film Growth at the Interface, Stress, and Gas Barrier Properties of Dyads,” ACS Applied Materials & Interfaces 10 (2018): 7422–7434, 10.1021/acsami.7b14916. 29338170

[97] M. Mayer , “SIMNRA User's Guide. Report IPP 9/113”, https://mam.home.ipp.mpg.de/Report%20IPP%209‐113.pdf, 1997 (accessed February 06, 2026).

[98] N. Fairley , V. Fernandez , M. Richard‐Plouet , et al., “Systematic and Collaborative Approach to Problem Solving Using X‐ray Photoelectron Spectroscopy,” Applied Surface Science Advances 5 (2021): 100112, 10.1016/j.apsadv.2021.100112.

[99] R. Ahlrichs , M. Bär , M. Häser , H. Horn , and C. Kölmel , “Electronic Structure Calculations on Workstation Computers: The Program System Turbomole,” Chemical Physics Letters 162 (1989): 165–169, 10.1016/0009-2614(89)85118-8.

[100] S. G. Balasubramani , G. P. Chen , S. Coriani , et al., “TURBOMOLE: Modular Program Suite for Ab Initio Quantum‐chemical and Condensed‐matter Simulations,” The Journal of Chemical Physics 152 (2020): 184107, 10.1063/5.0004635. 32414256 PMC7228783

[101] J. P. Perdew , K. Burke , and M. Ernzerhof , “Generalized Gradient Approximation Made Simple,” Physical Review Letters 77 (1996): 3865–3868, 10.1103/PhysRevLett.77.3865. 10062328

[102] C. Adamo and V. Barone , “Toward Reliable Density Functional Methods Without Adjustable Parameters: The PBE0 Model,” The Journal of Chemical Physics 110 (1999): 6158–6170, 10.1063/1.478522.

[103] F. Weigend and R. Ahlrichs , “Balanced Basis Sets of Split Valence, Triple Zeta Valence and Quadruple Zeta Valence Quality for H to Rn: Design and Assessment of Accuracy,” Physical Chemistry Chemical Physics 7 (2005): 3297, 10.1039/b508541a. 16240044

[104] F. Weigend , “Accurate Coulomb‐fitting Basis Sets for H to Rn,” Physical Chemistry Chemical Physics 8 (2006): 1057, 10.1039/b515623h. 16633586

[105] G. Kresse and J. Hafner , “ *Ab Initio* Molecular Dynamics for Liquid Metals,” Physical Review B 47 (1993): 558–561, 10.1103/PhysRevB.47.558. 10004490

[106] G. Kresse and J. Furthmüller , “Efficiency of Ab‐initio Total Energy Calculations for Metals and Semiconductors Using a Plane‐wave Basis Set,” Computational Materials Science 6 (1996): 15–50, 10.1016/0927-0256(96)00008-0.

[107] G. Kresse and J. Furthmüller , “Efficient Iterative Schemes for *Ab Initio* Total‐energy Calculations Using a Plane‐wave Basis Set,” Physical Review B 54 (1996): 11169–11186, 10.1103/PhysRevB.54.11169. 9984901

[108] S. Grimme , J. Antony , S. Ehrlich , and H. Krieg , “A Consistent and Accurate *Ab Initio* Parametrization of Density Functional Dispersion Correction (DFT‐D) for the 94 Elements H‐Pu,” The Journal of Chemical Physics 132 (2010): 154104, 10.1063/1.3382344. 20423165

[109] P. E. Blöchl , “Projector Augmented‐wave Method,” Physical Review B 50 (1994): 17953. 10.1103/physrevb.50.17953 9976227

[110] H. J. Monkhorst and J. D. Pack , “Special Points for Brillouin‐zone Integrations,” Physical Review B 13 (1976): 5188–5192, 10.1103/PhysRevB.13.5188.

[111] A. L. Wilkins , P. J. Watkinson , and K. M. Mackay , “Aspects of Germanium‐73 Nuclear Magnetic Resonance Spectroscopy,” Journal of the Chemical Society, Dalton Transactions 16 (1987): 2365–2372, 10.1039/dt9870002365.

[112] A. Tzalmona , “Measurement of the ^73^Ge‐proton Spin‐spin Coupling in Ge(CH_3_)_4_ ,” Molecular Physics 7 (1964): 497–498, 10.1080/00268976300101291.

[113] J. Kaufmann and W. Sahm , “ ^73^Ge Nuclear Magnetic Resonance Studies,” Zeitschrift für Naturforschung A 26 (1971): 1384–1389, 10.1515/zna-1971-0902.

[114] C. S. Weinert , “G73e Nuclear Magnetic Resonance Spectroscopy of Germanium Compounds,” ISRN Spectroscopy 2012 (2012): 1–18, 10.5402/2012/718050.

