# Supplementary material for: A Liquid Ge(IV) Precursor for Low Temperature Plasma Enhanced Atomic Layer Deposition of Germanium Oxide Thin Films
Source: Small. 2026 Feb 19;22(20):e11982. doi: 10.1002/smll.202511982 (PMC13054212; doi:10.1002/smll.202511982)
Supplement: Supplementary file 1 — The authors have cited additional references within the Supporting Information [57, 59, 64, 111, 112, 113, 114]. Deposition Number 2485525 contains the supplementary crystallographic data for [Ge(DMP)4]. This data is provided free of charge by the joint Cambridge Crystallographic Data Centre and Fachinformationszentrum Karlsruhe Access Structures service. Supporting File: smll72769‐sup‐0001‐SuppMat.pdf. [file SMLL-22-e11982-s001.pdf]

## Supporting Information

# A Liquid Ge(IV) Precursor for Low Temperature Plasma Enhanced Atomic Layer Deposition of Germanium Oxide Thin Films

Florian Preischel,[a,b] Karl Rönnby,[c] Martin Wilken[a] Jean-Pierre Glauber,[a,b] Samuel Froeschke,[b] Detlef Rogalla,[d] Thomas Gemming,[b] Alexey Popov,[b] Peter Dement,[b] Michael Nolan,[c] Anjana Devi*[a,b,e,f]

[a] Inorganic Materials Chemistry, Ruhr University Bochum, Universitätsstr. 150, 44801 Bochum, Germany
[b] Leibniz Institute for Solid State and Materials Research, Helmholtzstr. 20, 01069 Dresden, Germany
[c] Tyndall National Institute, Lee Maltings, University College Cork, Cork T12 R5CP, Ireland
[d] RUBION, Ruhr University Bochum, Universitätsstr. 150, 44801 Bochum, Germany
[e] Fraunhofer Institute for Microelectronic Circuits and Systems (IMS), Finkenstr. 61, Duisburg, Germany
[f] Chair of Materials Chemistry, TU Dresden, Bergstr. 66, 01069 Dresden, Germany

## Contents

**Characterization of [Ge(DMP)$_4$] (NMR, 2D NMR, EA, SC-XRD, TGA) and DFT Structures of Heteroleptic Variants** **2**

**PEALD Process Development and Characterization of GeO$_x$ Thin Films (XRR, GI-XRD, RBS/NRA, DFT, XPS, Refractive Index, UV/Vis, TEM, QCM)** **10**

**References** **16**

## Characterization of [Ge(DMP)$_4$] (NMR, 2D NMR, EA, SC-XRD, TGA) and DFT Structures of Heteroleptic Variants

The spectroscopic purity of [Ge(DMP)$_4$] was examined using $^1$H and $^{13}$C NMR spectroscopy. The $^1$H NMR spectrum (**Figure 1a**) shows four distinct peaks that can be assigned to the protons of [Ge(DMP)$_4$] as follows: peak A is a triplet at 2.24 ppm with an integral of 8 representing the protons of the $CH_2$ group at the amino function. In contrast, the protons of the two $CH_3$ groups appear as a singlet at 2.15 ppm (peak B, 24H) because they do not have any neighboring protons. Meanwhile, peak C with an integral of 2 is assigned to the protons of the $CH_2$ group in the middle of the propyl chain and appears as a multiplet at 1.63 ppm. The multiplet peak D (8H) is most upfield-shifted at 0.86 ppm, clearly indicating the shielding effect of the electropositive Ge center and thus belongs to the protons of the $CH_2$ group directly adjacent to the Ge. The splitting of peaks C and D is noteworthy, as from the $J_{HH}$ coupling alone, they would be expected to appear as a quintet and a triplet, respectively. For clarity, a magnified view of these two peaks is provided in **Figure S1**. The observed splitting can be ascribed to $^2J_{GeH}$ and $^3J_{GeH}$ coupling, which is often observed in Ge alkyl compounds due to the NMR-active $^{73}$Ge nucleus with a nuclear spin of 9/2.[1,2,3,4]

Analogously, in the $^{13}$C NMR spectrum (**Figure 1b**), peaks d (63.53 ppm), c (23.98 ppm) and a (10.66 ppm), each with an integral of 4, belong to the carbons of the propyl chain, whereby those closer to the Ge center are more upfield shifted. Peak b with an integral of 8 at 45.69 ppm belongs to the carbons of the two $CH_3$ groups.

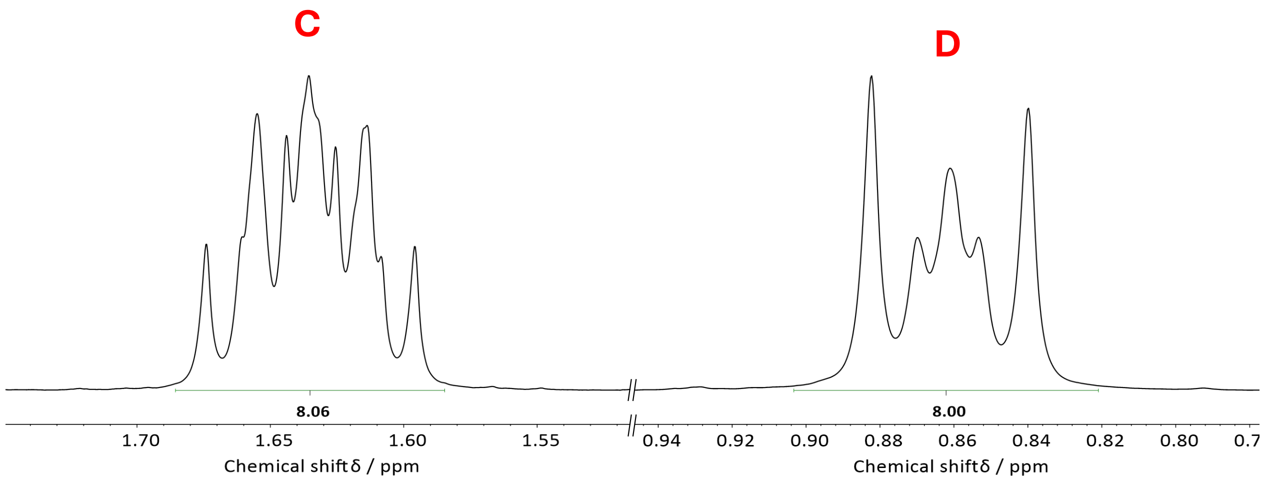


**Figure S1.** Magnified version of peaks C and D of the $^1$H NMR spectrum of [Ge(DMP)$_4$].

To further verify the peak assignments from the 1D $^{1}$H and $^{13}$C NMR spectra (**Figure 1**), 2D spectra were recorded to evaluate the $J_{HH}$ couplings (*via* $^{1}$H,$^{1}$H-COSY NMR) as well as $^{2}J_{CH}$ and $^{3}J_{CH}$ couplings (*via* $^{1}$H,$^{13}$C-HMBC NMR). The spectra shown in **Figures S2** and **S3** along with the derived couplings confirm the previous peak assignments.

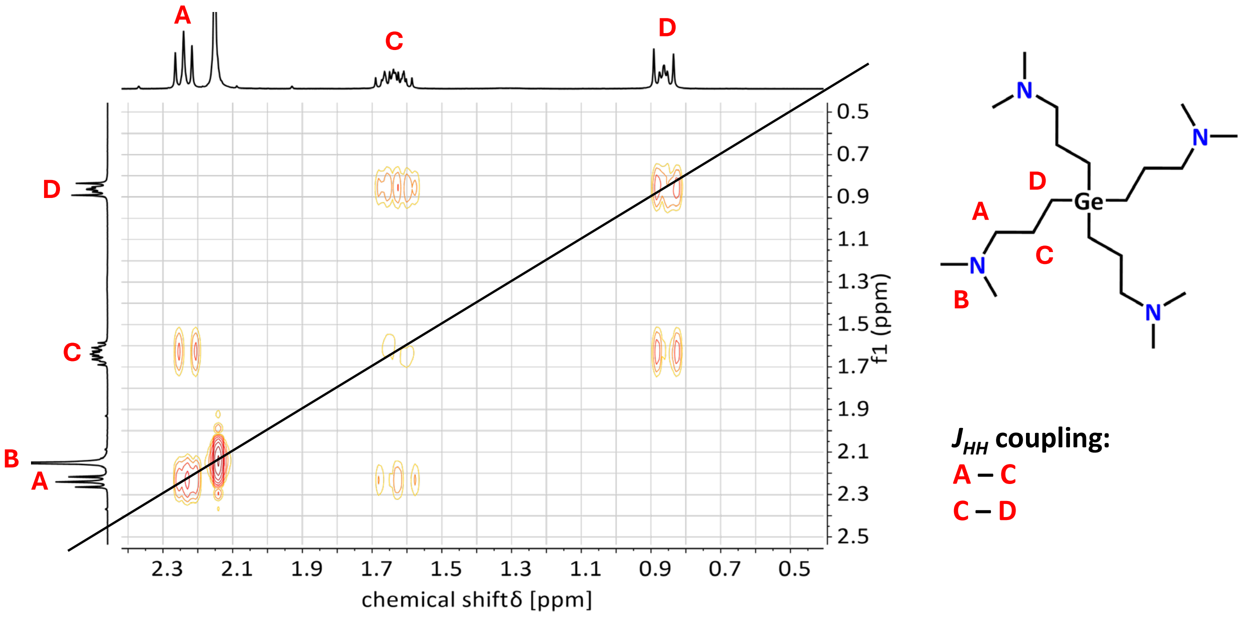


**Figure S2.** $^{1}$H,$^{1}$H-COSY NMR spectrum of [Ge(DMP)$_4$] with assignment of the peaks and the observed $J_{HH}$ couplings.

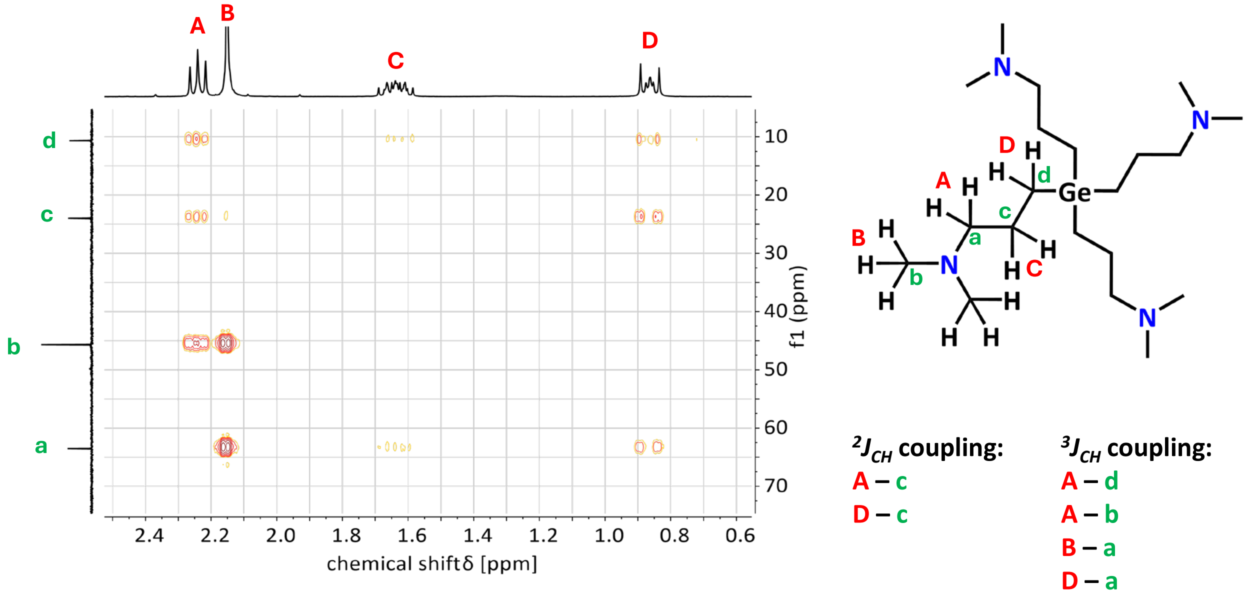


**Figure S3.** $^{1}$H,$^{13}$C-HMBC NMR spectrum of [Ge(DMP)$_4$] with peak assignments and observed $^{2}J_{CH}$ and $^{3}J_{CH}$ couplings.

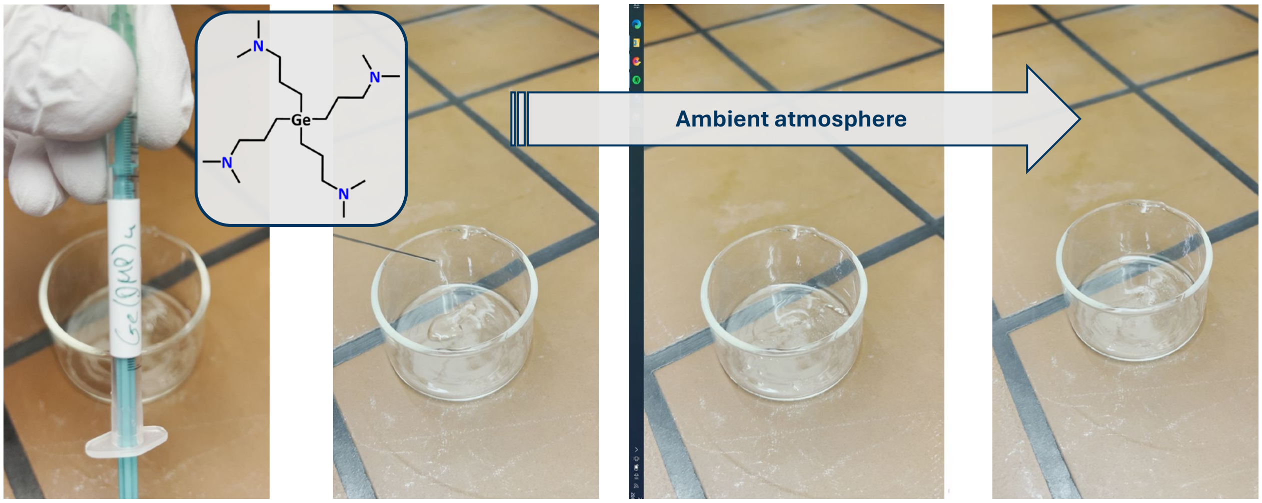


**Figure S4.** Image series, showing the exposure of pure [Ge(DMP)$_4$] to the ambient atmosphere over a period of approximately one minute.

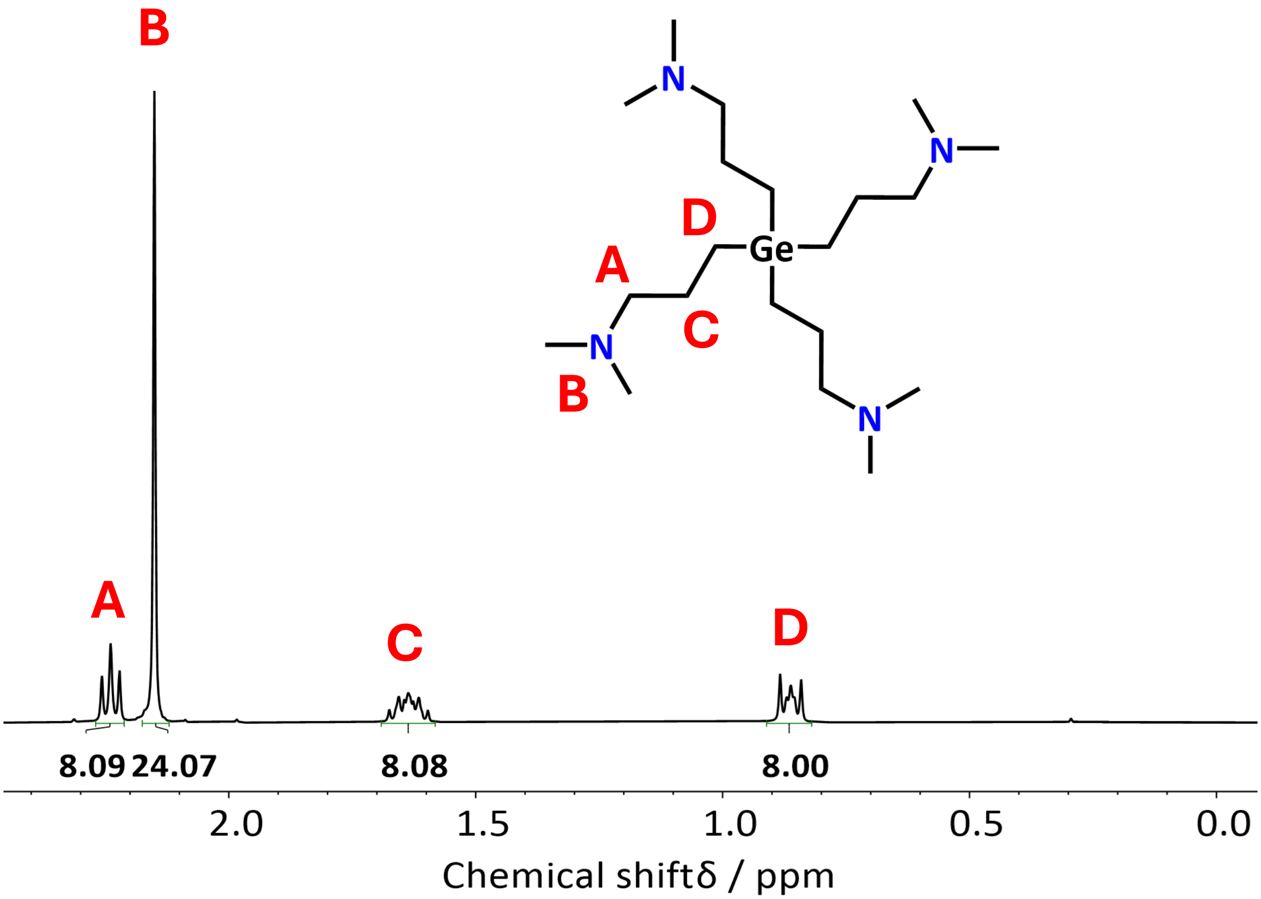


**Figure S5.** $^1$H NMR of [Ge(DMP)$_4$] after use in PEALD at a precursor bubbler temperature of 120 °C.

**Table S1.** Elemental analysis of [Ge(DMP)$_4$].

| Element | Calc. (%) | Found (%) |
|---|---|---|
| C | 57.57 | 57.42 |
| H | 11.60 | 11.64 |
| N | 13.43 | 13.44 |
| Ge | 17.41 | 17.32 |

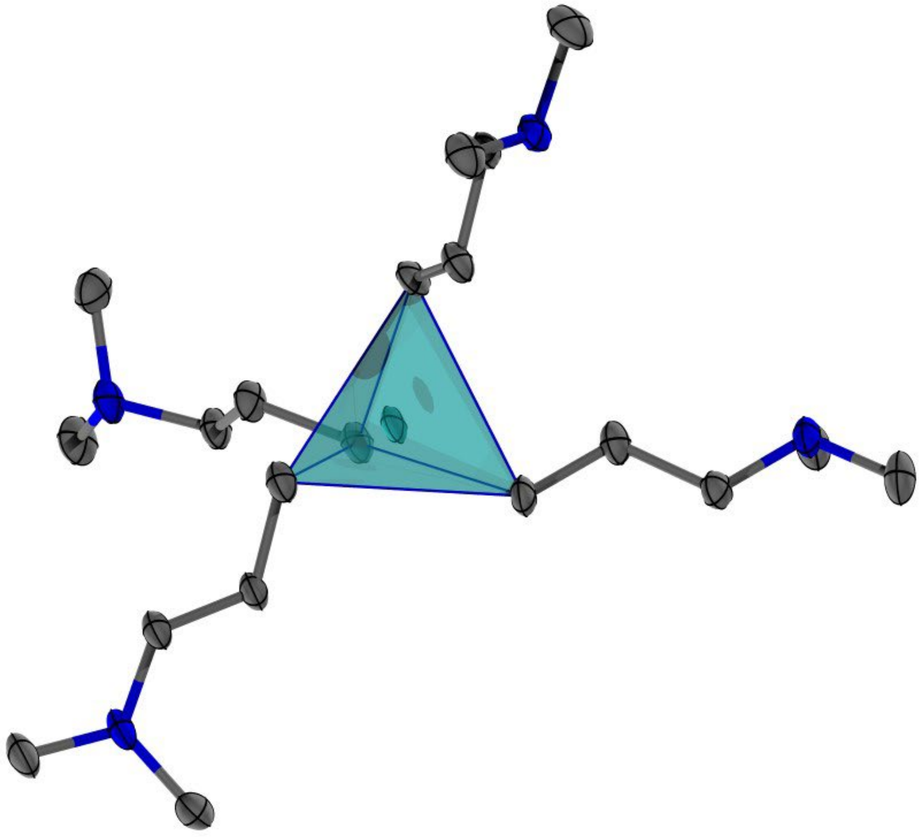

**Figure S6.** SC-XRD structure of [Ge(DMP)$_4$] with the tetrahedral coordination around the central Ge atom.

**Table S2.** Selected bond lengths and angles in the SC-XRD structure of [Ge(DMP)$_4$].

| Bond length (Å) | | Bond angle (°) | |
|---|---|---|---|
| Ge–C1 | 1.966(3) | C1–Ge–C2 | 110.24(11) |
| Ge–C2 | 1.970(2) | C1–Ge–C3 | 107.48(15) |
| Ge–C3 | 1.966(3) | C1–Ge–C4 | 110.66(11) |
| Ge–C4 | 1.970(2) | C2–Ge–C3 | 110.66(11) |
| | | C2–Ge–C4 | 107.57(15) |
| | | C3–Ge–C4 | 110.24(11) |

**Table S3.** Crystallographic data of [Ge(DMP)$_4$] (CCDC Deposition Number: 2485525)

| Parameter | Value |
|---|---|
| Empirical formula | $C_{20}H_{48}GeN_4$ |
| Formula weight (g mol$^{-1}$) | 417.244 |
| Temperature (K) | 102.0(8) |
| Crystal system | monoclinic |
| Space group | C2/c |
| a, b, c (Å) | 17.3603(2), 8.54340(10), 16.8872(3) |
| α, β, γ (°) | 90, 91.130(1), 90 |
| Volume (Å$^3$) | 2504.15(6) |
| Z (number of molecules in the unit cell) | 4 |
| $\rho_{calc}$ (g cm$^{-3}$) | 1.107 |
| $\mu$ (mm$^{-1}$) | 1.711 |
| F(000) | 909.7 |
| Crystal size (mm$^3$) | 0.1 × 0.1 × 0.2 |
| Radiation | Cu Kα (λ = 1.54184) |
| 2Θ range for data collection (°) | 10.192 to 153.48 |
| Index ranges | –21 ≤ h ≤ 21, –10 ≤ k ≤ 9, –21 ≤ l ≤ 20 |

| | |
|---|---|
| Reflections collected | 11647 |
| Independent reflections | 2479 [$R_{int}$ = 0.1213, $R_{sigma}$ = 0.0652] |
| Data/restraints/parameters | 2479/0/118 |
| Goodness-of-fit on $F^2$ | 1.007 |
| Final R indices [$I \geq 2\sigma(I)$] | $R_1$ = 0.0461, $wR_2$ = 0.1346 |
| Final R indices [all data] | $R_1$ = 0.0481, $wR_2$ = 0.1368 |
| Largest diff. peak/hole ($e$Å$^{-3}$) | 0.75/–0.66 |

It was necessary to crystallize [Ge(DMP)$_4$] under dry-ice cooling, while handling of the single crystal and transferring it to the microscope had to be carried out under $N_2$ cooling. Despite these preparatory steps, the outer part of the single crystal melted during the transfer, resulting in reduced crystal quality, which explains the higher $R_{int}$ value of 0.1213.

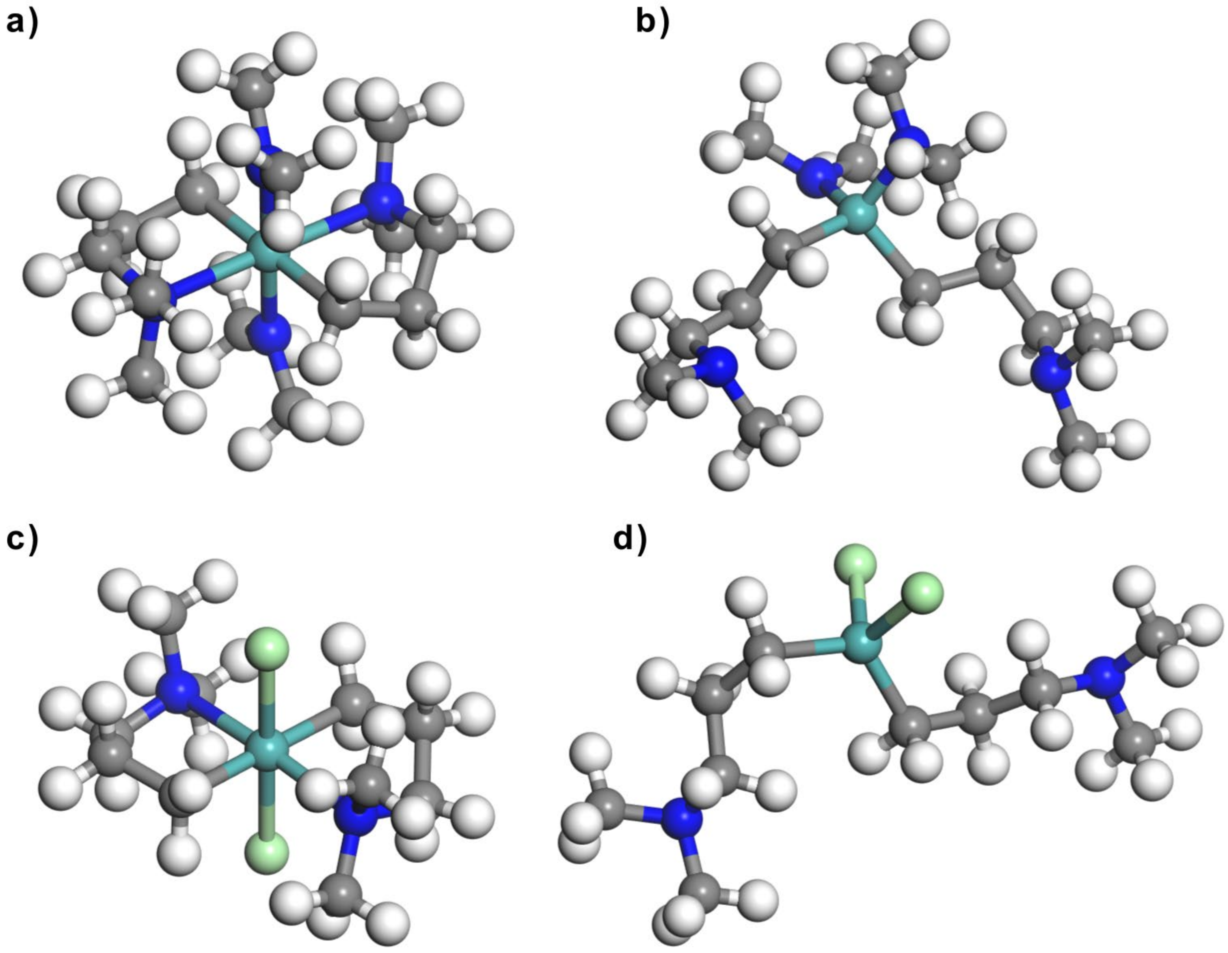


**Figure S7.** Optimized geometries for [Ge(DMP)$_2$(NMe$_2$)$_2$] in **a)** chelated and **b)** non-chelated forms, and [Ge(DMP)$_2$Cl$_2$] in **c)** chelated and **d)** non-chelated forms. Color scheme: turquoise for Ge, grey for C, blue for N, light green for Cl, and white for H.

**Table S4.** Thermal properties of [Ge(DMP)$_4$] as derived from TG measurements with various heating rates and from a stepped isothermal TG.

| **Thermal Properties** | **[Ge(DMP)$_4$]** | |
|---|---|---|
| | 5 K/min | 20 K/min |
| Onset (°C) | 178 | 204 |
| Step temperature (°C) | 260 | 304 |
| $T_{1Torr}$ (°C) | 183 | |
| $r_v$ at $T_{1Torr}$ (µg min$^{-1}$ cm$^{-2}$) | 329 | |

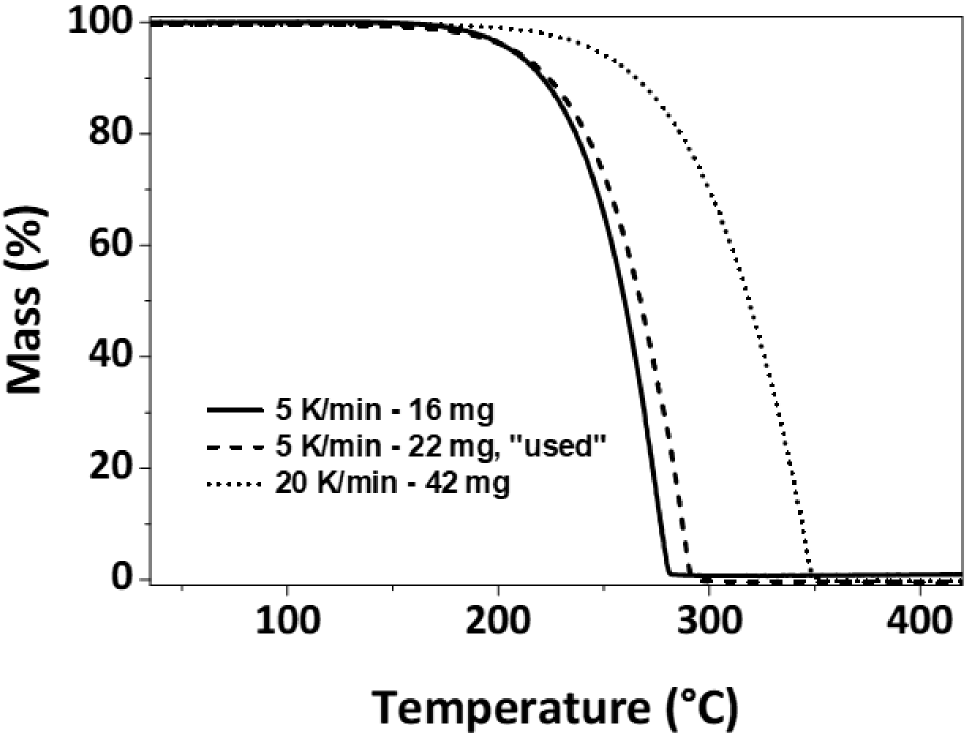


**Figure S8.** TG curves of [Ge(DMP)$_4$] measured with different heating rates and mass loadings (solid and dotted curves) as well as after prolonged heating to 120 °C for PEALD experiments (dashed curve, "used"). The shift in evaporation of the "used" precursor to slightly higher temperatures can be attributed to the slightly higher mass load during this TG experiment.

**Table S5.** Volatilization rates ($r_v$) and molar volatilization rates ($r_{vm}$) of [Ge(DMP)$_4$] were determined from a stepped *iso*thermal TG, with a comparison to the respective values for [Sn(DMP)$_4$].

| Temperature (°C) | $r_v$ (µg min$^{-1}$ cm$^{-2}$) | | $r_{vm}$ (µmol min$^{-1}$ cm$^{-2}$) | |
|---|---|---|---|---|
| | [Ge(DMP)$_4$] | [Sn(DMP)$_4$][5] | [Ge(DMP)$_4$] | [Sn(DMP)$_4$][5] |
| 110 | - | 6.0 | - | 0.013 |
| 130 | 24 | 14.1 | 0.059 | 0.030 |
| 140 | 37 | - | 0.088 | - |
| 150 | 65 | 51.5 | 0.156 | 0.111 |
| 160 | 111 | - | 0.265 | - |
| 170 | 185 | - | 0.444 | - |
| 180 | 299 | - | 0.718 | - |
| 190 | 465 | - | 1.114 | - |

## PEALD Process Development and Characterization of $GeO_x$ Thin Films (XRR, GI-XRD, RBS/NRA, DFT, XPS, Refractive Index, UV/Vis, TEM, QCM)

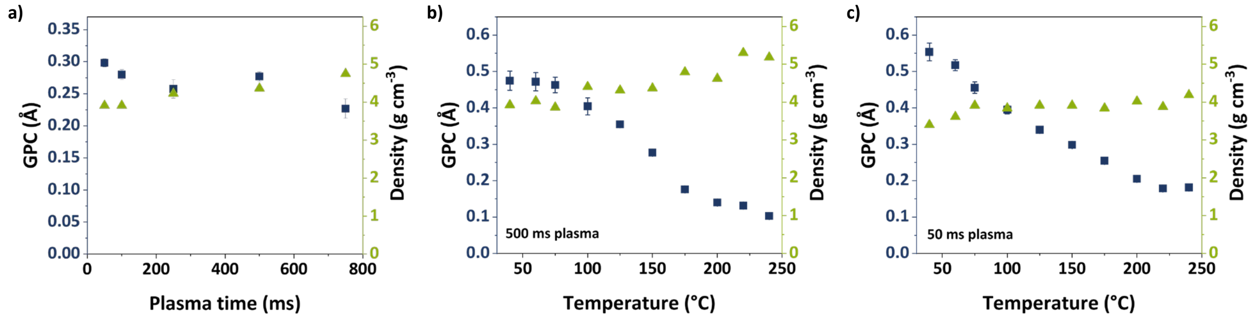


**Figure S9.** XRR-derived GPC and density of $GeO_x$ thin films deposited on Si with **a)** varying plasma pulse times at a substrate temperature of 150 °C, as well as at varying temperatures with fixed plasma times of **b)** 500 ms and **c)** 50 ms.

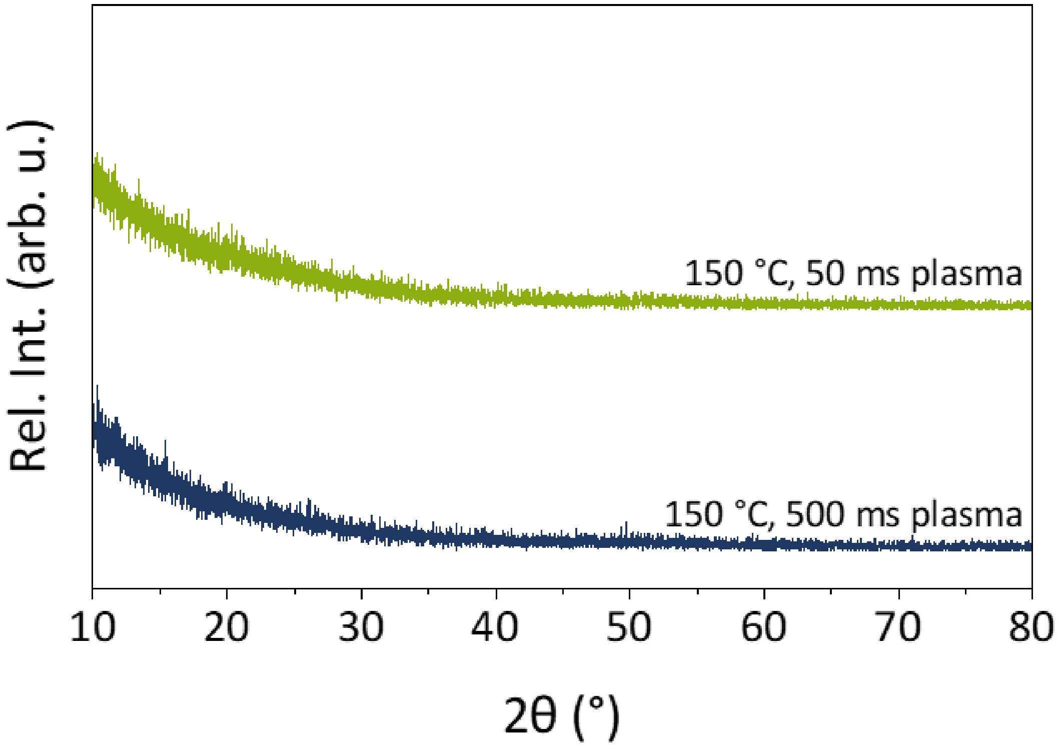


**Figure 10.** GI-XRD of $GeO_2$ thin films deposited at 150 °C on Si with plasma durations of 500 ms (blue spectrum, ca. 14 nm film thickness) and 50 ms (green spectrum, ca 15 nm film thickness).

**Table S6.** Composition of $GeO_x$ thin films deposited on Si at 150 °C with different plasma pulse durations using $[Ge(DMP)_4]$, as determined by RBS/NRA.

| Plasma (ms) | O (at%[a]) | Ge (at%[a]) | O/Ge ratio[b] | C (at%[a]) | N (at%[a]) |
|---|---|---|---|---|---|
| **750** | 50.0 | 43.3 | 1.15 | 2.4 | 4.3 |
| **500** | 55.9 | 39.4 | 1.42 | 2.7 | 2.0 |
| **250** | 50.3 | 41.0 | 1.23 | 2.0 | 6.7 |
| **100** | 64.2 | 31.8 | 2.02 | 1.1 | 3.0 |
| **50** | 63.3 | 31.6 | 2.00 | 1.4 | 3.7 |

[a)] Detection limits for C and N are $2.0\times10^{15}$ atoms per $cm^2$ and $8.0\times10^{15}$ atoms per $cm^2$, respectively. In all cases, a possible error of 1.5 at% must be assumed.

[b)] The O/Ge ratios have a relative error of ±2% and total normalization error of ±4%.

**Table S7.** Composition of $GeO_x$ thin films deposited on Si at varying temperatures and a plasma pulse time of 500 ms with $[Ge(DMP)_4]$, as determined by RBS/NRA.

| $T_{dep}$ (°C) | O (at%) | Ge (at%) | O/Ge ratio[b] | C (at%) | N (at%) |
|---|---|---|---|---|---|
| **40** | 64.0 | 30.1 | 2.13 | 2.9 | 3.0 |
| **60** | 65.0 | 31.7 | 2.05 | 3.2 | n.d. |
| **75** | 63.9 | 32.1 | 1.99 | 2.3 | 1.6 |
| **100** | 57.6 | 38.1 | 1.51 | 3.1 | 1.2 |
| **125** | 61.1 | 34.5 | 1.77 | 4.4 | n.d. |
| **150** | 55.9 | 39.4 | 1.42 | 2.7 | 2.0 |
| **175** | 52.5 | 37.1 | 1.41 | 7.0 | 3.4 |
| **200** | 52.9 | 40.9 | 1.29 | 6.2 | n.d. |
| **225** | 51.4 | 42.9 | 1.20 | 5.7 | n.d. |
| **240** | 55.1 | 41.6 | 1.33 | 3.3 | n.d. |
| **240, Δ[c]** | 49.0 | 34.4 | 1.42 | 13.8 | 2.8 |

[a)] n.d = not detected species with detection limits for C and N of $2.0\times10^{15}$ atoms per $cm^2$ and $8.0\times10^{15}$ atoms per $cm^2$, respectively. In all cases, a possible error of 1.5 at% must be assumed.

[b)] The O/Ge ratios have a relative error of ±2% and an overall normalization error of ±4%.

[c)] This entry shows the values obtained for the sample deposited at 240 °C after undergoing a post-deposition annealing step at 400 °C for 20 min under ambient conditions.

**Table S8.** Composition of $GeO_x$ thin films deposited on Si at various temperatures and a plasma pulse time of 50 ms with $[Ge(DMP)_4]$, as determined by RBS/NRA.

| $T_{dep}$ (°C) | O (at%[a]) | Ge (at%[a]) | O/Ge ratio[b] | C (at%[a]) | N (at%[a]) |
|---|---|---|---|---|---|
| **40** | 52.6 | 28.0 | 1.88 | 11.6 | 7.7 |
| **60** | 60.0 | 29.7 | 2.02 | 5.3 | 5.0 |
| **75** | 64.6 | 31.5 | 2.05 | 1.6 | 2.2 |
| **100** | 64.6 | 30.8 | 2.10 | 1.4 | 3.2 |
| **125** | 66.3 | 32.0 | 2.07 | 1.2 | 0.4 |
| **150** | 63.3 | 31.6 | 2.00 | 1.4 | 3.7 |
| **175** | 64.2 | 323 | 1.99 | 1.5 | 2.0 |
| **200** | 64.4 | 29.9 | 2.16 | 0.6 | 5.1 |
| **225** | 65.7 | 32.4 | 2.03 | 1.2 | 0.7 |
| **240** | 61.1 | 32.6 | 1.87 | 1.5 | 4.8 |

[a)] Detection limits for C and N are $2.0\times10^{15}$ atoms per $cm^2$ and $8.0\times10^{15}$ atoms per $cm^2$, respectively. In all cases, a possible error of 1.5 at% must be assumed.
[b)] The O/Ge ratios have a relative error of ±2% and a total normalization error of ±4%.

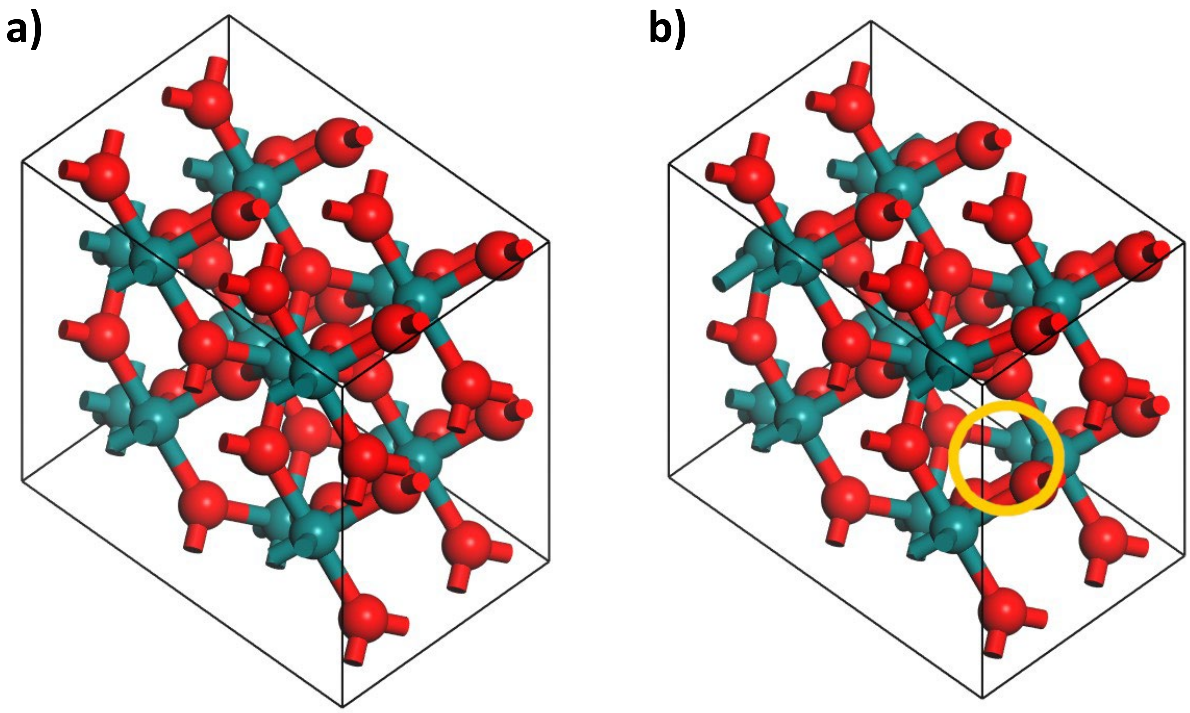


**Figure S11.** Relaxed structure of a) bulk $GeO_2$ and b) bulk $GeO_2$ with an oxygen vacancy created by reaction with $^3O$. The vacancy site is highlighted by the yellow circle. Turquoise spheres represent Ge, and red ones O.

**Figure S12** compares the XPS survey spectra of as-introduced and sputtered surfaces of a $GeO_x$ thin film deposited at 240 °C with a 500 ms plasma pulse and high-resolution scans of the Ge 3d region. In the survey spectra, the N 1s and C 1s regions are marked in grey, showing the absence of N and a minor C signal in the as-introduced surface. Upon sputtering, the C signal disappears, indicating that the detected C is attributable to adventitious carbon. However, the Ge 3d scan shows that the $Ar^+$-sputter step significantly influences the oxidation states of the Ge, as $Ge^{4+}$ is completely removed, while the $Ge^{2+}$ and $Ge^0$ peaks are increased.

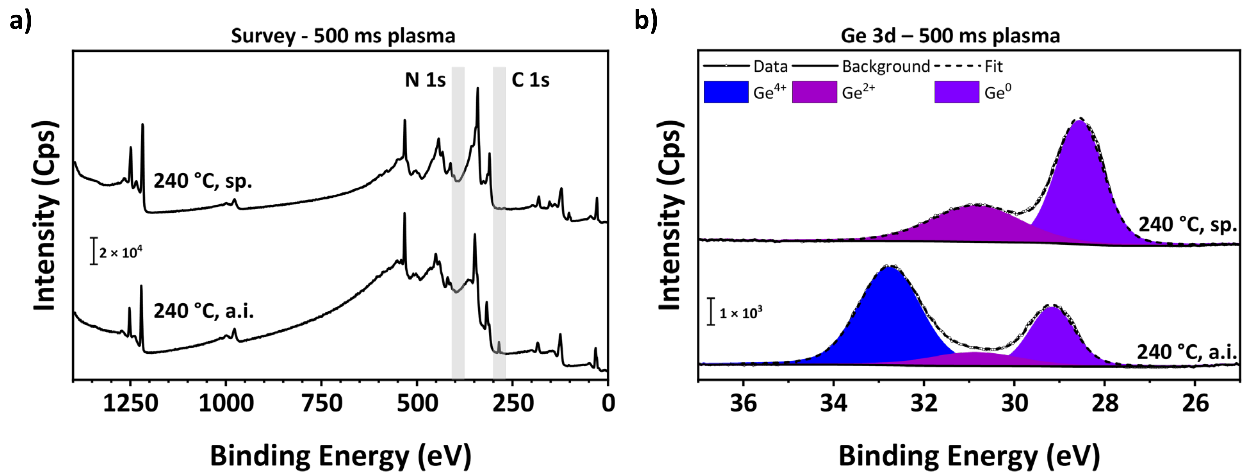


**Figure S12.** XPS recordings showing a) survey spectra and b) high resolution spectra of the Ge 3d core level region of as-introduced (bottom) and sputtered (top) $GeO_x$ thin films deposited on Si with [Ge(DMP)$_4$] at 240 °C using 500 ms plasma pulses.

High-resolution scans of the O 1s core level show two peaks, corresponding to O–Ge–O and Ge–O (**Figure S12**). This confirms the presence of $GeO_2$ and GeO in the films, as seen in the Ge 3d region. The O–Ge–O signal appears between 531.5 eV – 532.0 eV, and the Ge–O signal between 529.9 eV – 530.4 eV, which is consistent with reported values for other ALD deposited $GeO_x$ films.[6,7]

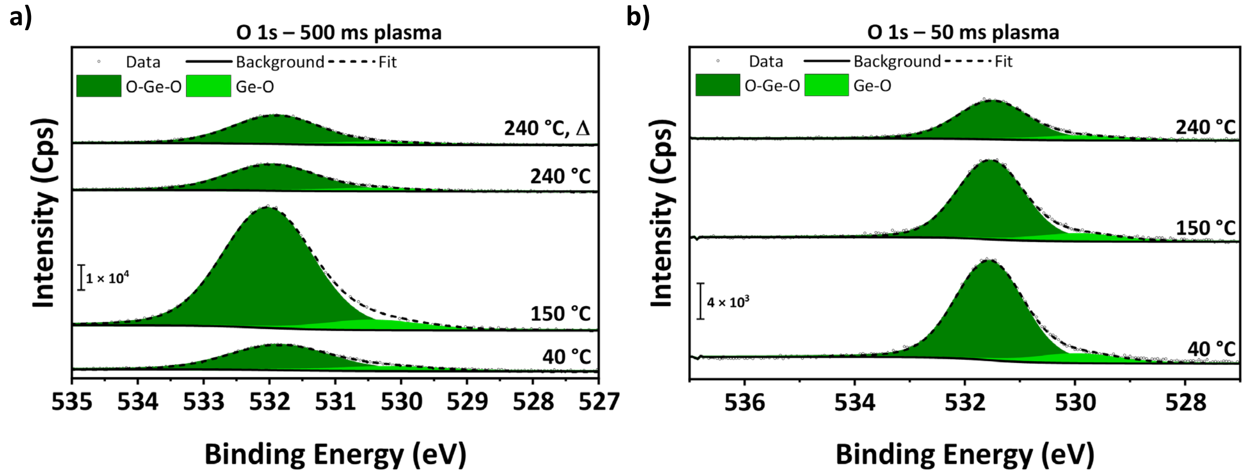


**Figure S13.** High-resolution XPS spectra of the O 1s core level regions of as-introduced $GeO_x$ thin films deposited on Si using [Ge(DMP)$_4$] at varying temperatures. Samples in **a)** were deposited with 500 ms plasma pulses, and the uppermost sample was annealed for 20 min at 400 °C under ambient conditions. Samples in **b)** were deposited with 50 ms plasma pulses.

**Table S9.** Relative shares of $Ge^{4+}$, $Ge^{2+}$, and $Ge^0$ as determined by a fit of high-resolution XPS spectra of the Ge 3d core level region from as-introduced $GeO_x$ thin films deposited with [Ge(DMP)$_4$] at various temperatures and plasma exposure times of 500 ms and 50 ms. The last column lists the $GeO_x$ stoichiometry calculated from these shares.

| Temperature (°C) | Plasma (ms) | Ge (%) | | | $GeO_x$ |
|---|---|---|---|---|---|
| | | $Ge^{4+}$ | $Ge^{2+}$ | $Ge^0$ | |
| **40** | **500** | 88.16 | 11.84 | - | 1.88 |
| **150** | **500** | 82.49 | 8.05 | 9.46 | 1.73 |
| **240** | **500** | 61.69 | 11.48 | 26.83 | 1.35 |
| **240, Δ[a]** | **500** | 71.76 | 10.49 | 17.75 | 1.54 |
| **40** | **50** | 95.07 | 4.93 | - | 1.95 |
| **150** | **50** | 94.90 | 5.10 | - | 1.95 |
| **240** | **50** | 79.09 | 15.27 | 5.64 | 1.74 |

[a)] This entry shows the values obtained for the sample deposited at 240 °C after treatment by a post-deposition annealing step at 400 °C for 20 min under ambient conditions.

**Table S10.** Refractive indices at a wavelength of 500 nm, derived from ellipsometry measurements. Additionally, the O/Ge ratios determined by RBS are shown.

| Temperature (°C) | Plasma (ms) | O/Ge | Refractive index *n* |
|---|---|---|---|
| **40** | **500** | 2.13 | 1.65 |
| **150** | **500** | 1.42 | 1.98 |
| **240** | **500** | 1.33 | 1.66 |
| **40** | **50** | 1.88 | 1.62 |
| **150** | **50** | 2.00 | 1.62 |
| **240** | **50** | 1.87 | 1.82 |

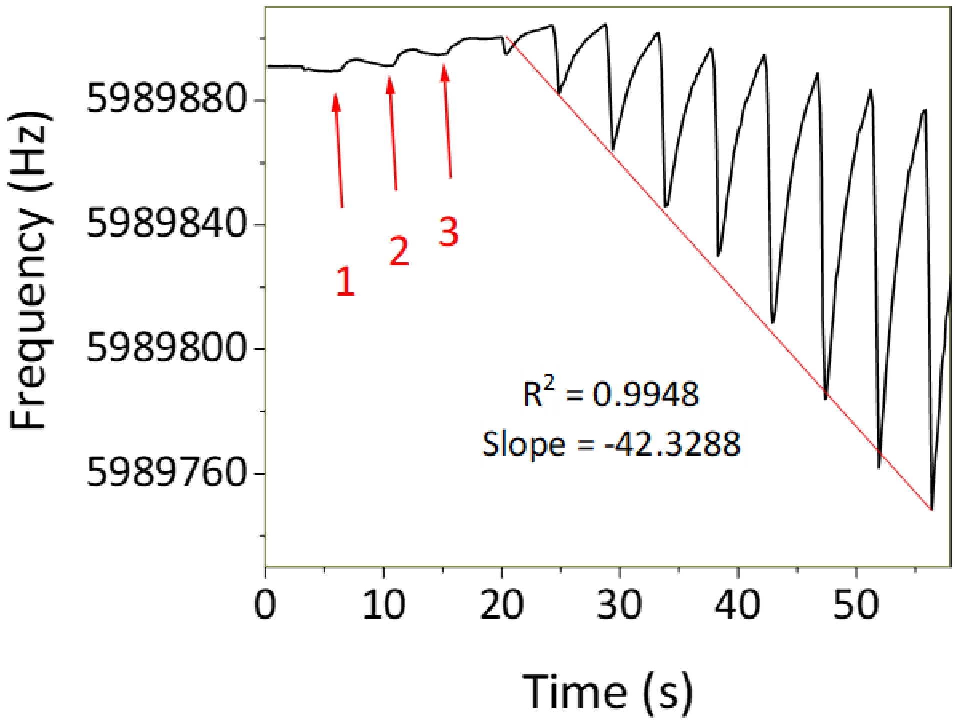


**Figure S14.** Recorded QCM frequency data of the initial PEALD cycles on an Au-coated quartz crystal, including a linear fit of the local frequency minima (red line). The quartz crystal was pretreated with 10×150 ms of plasma exposure prior to the PEALD cycles.

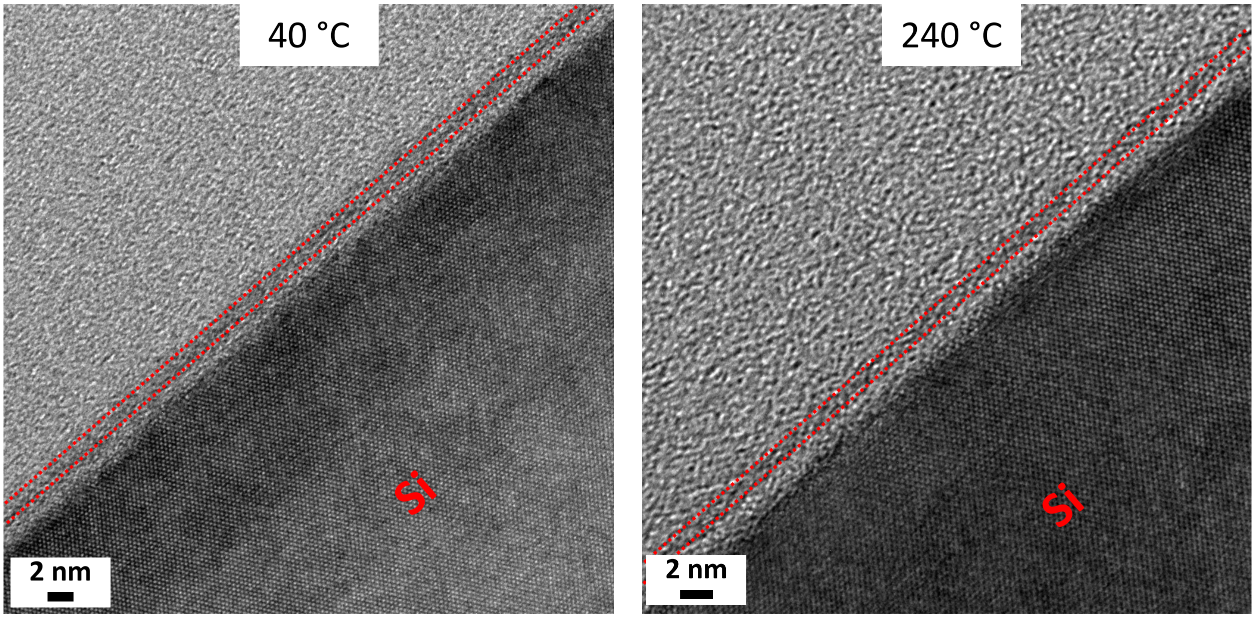


**Figure S15.** HRTEM images of $GeO_2$ thin films deposited on Si with 8 cycles and 50 ms plasma durations at **a)** 40 °C and **b)** 240 °C. The substrate was pretreated with 10×150 ms of plasma exposure prior to $GeO_2$ deposition. The dotted red lines indicate the $GeO_2$ layer.

## References


[1] A. L. Wilkins, P. J. Watkinson, K. M. Mackay, "Aspects of germanium-73 nuclear magnetic resonance spectroscopy", *J. Chem. Soc., Dalton Trans.* **1987**, 2365.

[2] A. Tzalmona, "Measurement of the $^{73}$Ge-proton spin-spin coupling in $Ge(CH_3)_4$", *Mol. Phys.* **1964**, *7*, 497.

[3] J. Kaufmann, W. Sahm, "$^{73}$Ge Nuclear Magnetic Resonance Studies", *Z. Naturforsch. A* **1971**, *26*, 1384.

[4] C. S. Weinert, "G73e Nuclear Magnetic Resonance Spectroscopy of Germanium Compounds", *ISRN Spectrosc.* **2012**, *2012*, 1.

[5] L. Mai, D. Zanders, E. Subaşı, E. Ciftyurek, C. Hoppe, D. Rogalla, W. Gilbert, T. d. L. Arcos, K. Schierbaum, G. Grundmeier, C. Bock, A. Devi, "Low-Temperature Plasma-Enhanced Atomic Layer Deposition of Tin(IV) Oxide from a Functionalized Alkyl Precursor: Fabrication and Evaluation of $SnO_2$-Based Thin-Film Transistor Devices", *ACS Appl. Mater. Interfaces* **2019**, *11*, 3169.

[6] C. M. Yoon, I.-K. Oh, Y. Lee, J.-G. Song, S. J. Lee, J.-M. Myoung, H. G. Kim, H.-S. Moon, B. Shong, H.-B.-R. Lee, H. Kim, "Water-Erasable Memory Device for Security Applications Prepared by the Atomic Layer Deposition of $GeO_2$", *Chem. Mater.* **2018**, *30*, 830.

[7] H. Choi, C. Park, S. K. Lee, J. Y. Ryu, S. U. Son, T. Eom, T.-M. Chung, "New Heteroleptic Germanium Precursors for $GeO_2$ Thin Films by Atomic Layer Deposition", *ACS Omega* **2023**, *8*, 43759.
